# Nanoscale Covalent Organic Framework Confinement Enables Ultratough and Hyperelastic Hydrogels Applied as High‐Performance Electrolytes

**DOI:** 10.1002/advs.202522523

**Published:** 2026-03-19

**Authors:** Peiyao Yan, Wei Zhao, Hao Wang, Lu Wu, Yedong Ma, Binting Huang, Hantao Xu, Xueyan Liu, Siqi Liu, Xinyu Dong, Xiaoyang Zhang, Wei Zhai, Weiwei Zhang, Lin Xu, Dan Zhao, Chaobin He

**Affiliations:** ^1^ Department of Materials Science and Engineering National University of Singapore Singapore Singapore; ^2^ Department of Chemical and Biomolecular Engineering National University of Singapore Singapore Singapore; ^3^ State Key Laboratory of Advanced Technology for Materials Synthesis and Processing School of Materials Science and Engineering Wuhan University of Technology Wuhan China; ^4^ Key Laboratory for Advanced Materials and Institute of Fine Chemicals School of Chemistry and Molecular Engineering East China University of Science and Technology Shanghai China; ^5^ Department of Mechanical Engineering National University of Singapore Singapore Singapore; ^6^ Institute for Materials Research and Engineering (IMRE) Agency for Science Technology and Research (A∗STAR) Singapore Singapore

**Keywords:** aqueous Zn‐ion batteries, hydrogel electrolyte, nanoscale covalent organic frameworks, ultratough hydrogels

## Abstract

Hydrogels are widely applied in various fields, including energy storage and flexible electronics. However, their mechanical properties often fail to meet the requirements for long‐term and repeated deformation and full recovery. Achieving simultaneous improvement in the strength, toughness, and elasticity of hydrogels remains a significant challenge. Here, we report a nanoconfined polymerization strategy within the well‐designed, fully delaminated nanoscale covalent organic frameworks (nCOFs) that overcomes these trade‐offs. This approach yields hydrogels with an order increase in strength (from 0.3 to 3.2 MPa), a two orders enhancement in toughness (from 7.5 to 186 MJ/m^3^) and fracture energy (from 0.8 to 14.7 kJ m^−2^), and a very low‐hysteresis (∼93% energy recovery) recoverable deformation even after 2000% strain in the 100 cycles. The dense entanglements provide high strength and toughness, and nanochannel‐threaded crosslinking enables large elastic deformation. Furthermore, their robust architecture affords a fivefold improvement in puncture resistance, enabling application as dendrite‐inhibiting and durable quasi‐solid‐state Zn‐ion electrolytes. This bottom‐up toughening strategy based on the nano‐reactor nCOF structural design could guide the development of next‐generation tough hydrogels for applications such as flexible energy devices and related fields.

## Graphical abstract

Confinement by nanoscale covalent organic frameworks reinforces hydrogels, imparting exceptional mechanical strength and ionic conductivity for high‐performance electrolyte applications.

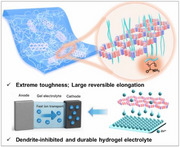

## 1 Introduction

Hydrogels, composed of crosslinked polymer networks swollen with water, have found extensive use in advanced technologies such as intelligent sensors [1], hydrogel electrolytes [2, 3], actuators [4], wound healing [5], and tissue engineering [6, 7]. Their mechanical properties, including strength, toughness, and elasticity, are critical determinants of both functionality and durability [8, 9, 10, 11]. However, pristine hydrogels generally suffer from poor mechanical robustness, motivating intensive efforts to develop reinforcement strategies. To date, these approaches can be broadly classified into two categories. The first relies on energy‐dissipative mechanisms, such as sacrificial bonds [12], microphase separation [13], dense entanglements [14, 15], typically employed in double‐network hydrogels. This design can significantly increase strength; however, it often compromises elasticity, as the rupture of sacrificial bonds and mechanical mismatch between networks lead to pronounced hysteresis during deformation [16, 17]. The second category focuses on topological structure design to achieve efficient stress distribution, as exemplified by slide‐ring hydrogels [10, 18], highly entangled hydrogels [8, 19], reversible pearl‐necklace building unit [20], and nanocomposites [21]. These systems usually display minimal hysteresis and excellent stress‐relaxation resistance under external loading [22, 23, 24]. However, such topologically engineered hydrogels typically display either large recoverable deformation (L/L_0_ ≥ 20) but low fracture energy (< 5 kJ/m^2^), or high energy absorption (up to ∼11 kJ/m^2^) with limited recoverable deformation (L/L_0_ < 5). Consequently, achieving a single hydrogel system that simultaneously delivers high strength, high toughness, and large‐deformation elasticity remains a formidable challenge in the field.

**FIGURE 1:**
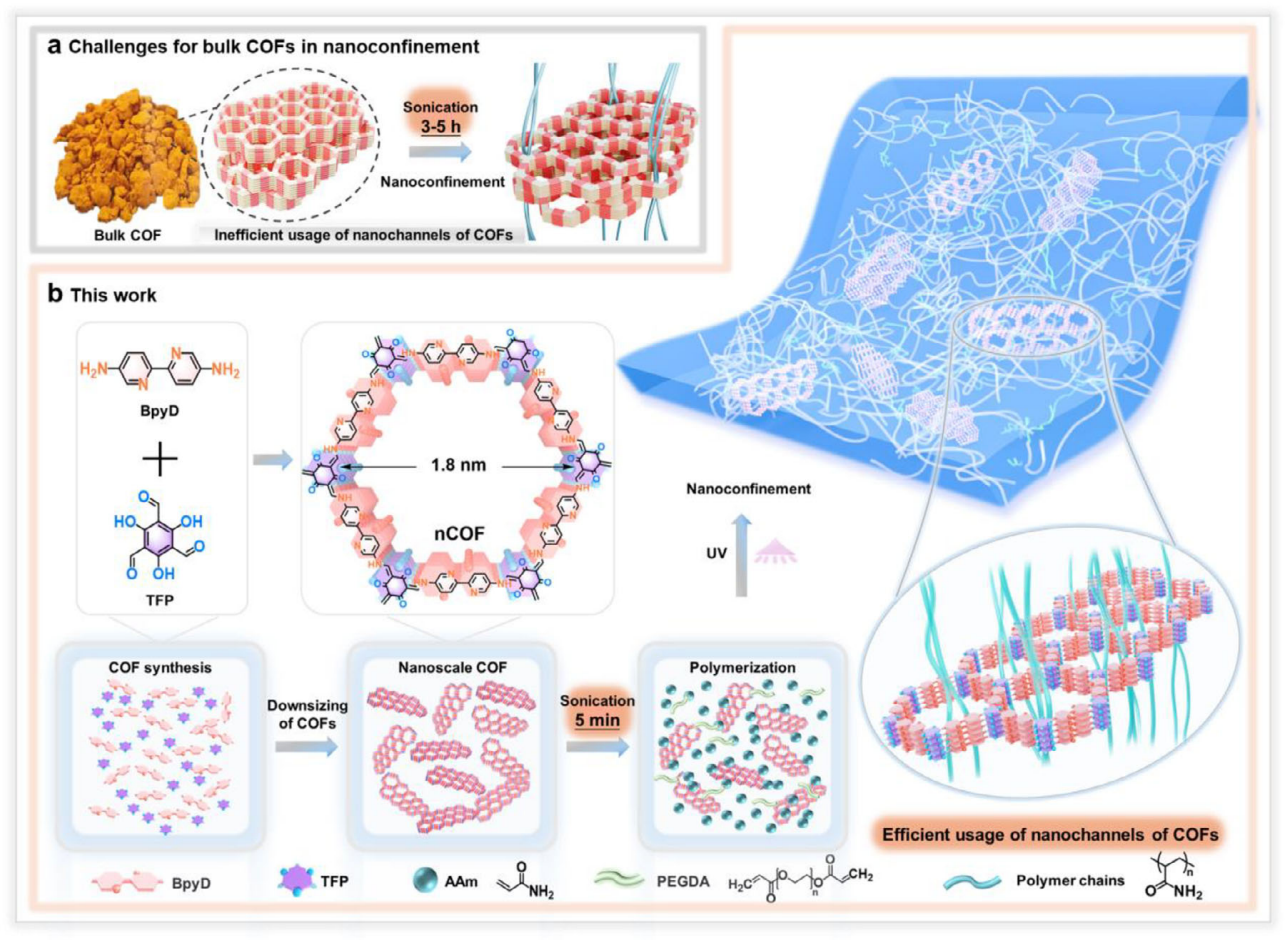
The synthetic routes to bulk and nanoscale COFs nanoconfined hydrogels. (a) Challenges associated with using bulk COFs in nanoconfinement. (b) Schematic illustration of the synthesis strategy in this work, featuring nanoscale COFs used for in situ polymerization, along with the structures of the monomers, polymer, and COF.

Nanoconfinement, wherein polymerization or molecular interactions are restricted within nanoscale domains, has emerged as a powerful strategy for tailoring the mechanical behavior of soft materials [25]. It can enhance stretchability [26], increase stiffness [27], and suppress crack propagation [28]. Covalent organic frameworks (COFs), with their highly ordered nanochannels, tunable chemistry, and rigid backbone, offer a promising platform to implement nanoconfinement within aqueous polymer networks [29, 30, 31, 32, 33]. For example, F. Yan et al. reported nanoconfined polymerization within COFs to produce hydrogels with reduced crack propagation and low hysteresis [28]. However, the mechanical reinforcement achieved remained limited, primarily due to the intrinsic characteristics of bulk COFs (bCOFs), which consist of polycrystalline domains with misaligned orientations [29, 30, 34] and tend to form microscale aggregates due to poor dispersion. These factors hinder effective utilization of their internal nanochannels (Figure 1a), resulting in suboptimal reinforcement. In contrast, nanoscale COFs (nCOFs), featuring fully delaminated layers, shortened diffusion paths, and accessible porous channels, offer a more favorable morphology for achieving efficient nanoconfinement [35, 36, 37, 38, 39]. Despite these advantages, the potential of nCOFs to enhance the mechanical properties of aqueous polymer systems remains largely unexplored.

Here, we propose a nanoconfined polymerization strategy enabled by fully delaminated nCOFs to overcome the intrinsic trade‐offs among strength, toughness, and elasticity in a single hydrogel system. Delaminated nCOF refers to ultrathin nanoscale COF structures with reduced interlayer stacking (∼5 nm thick), in contrast to conventional bulk COFs that form microscale aggregated particles with limited nanochannel accessibility. The well‐defined nanochannels of nCOFs provide confined reaction environments that guide polymer chain threading and entanglement during network formation. Compared to bulk COFs, these nCOFs exhibit superior dispersion, form stronger interfacial interactions, and contribute to significant mechanical enhancement through increased entanglement density and nanochannel‐threaded crosslink density. As a result, hydrogels exhibit outstanding mechanical properties, including improved strength, enhanced toughness, and hyperelasticity. Furthermore, the unique physicochemical properties of these hydrogels highlight their great potential as high‐performance quasi‐solid‐state electrolytes for Zn‐ion batteries.

## 2 Results

### 2.1 Preparation of nCOF‐Confined Hydrogels

**FIGURE 2:**
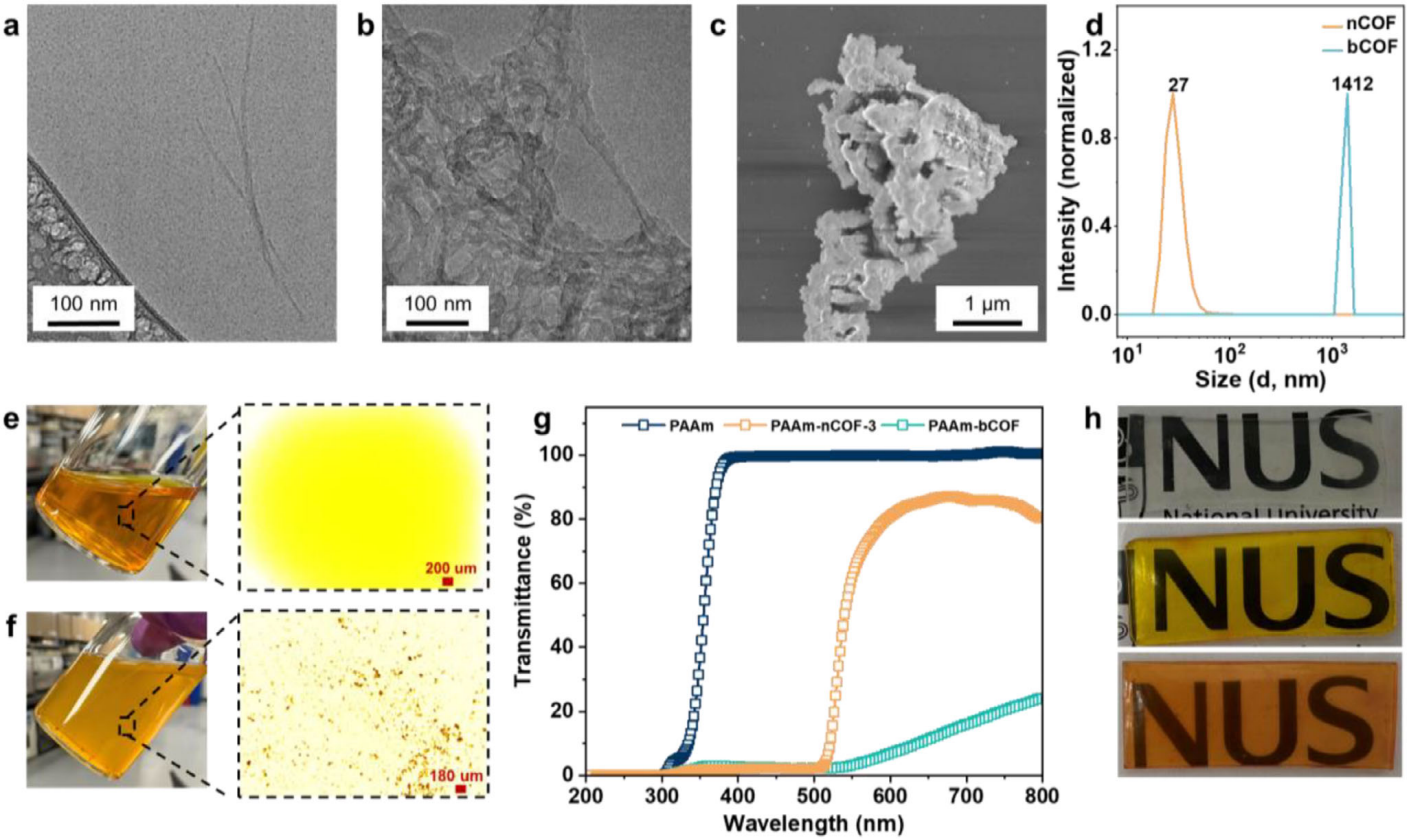
Studies on the dispersion of COFs in hydrogel precursors. (a) Cryo‐TEM image of nCOF (colloid state) (scale bar: 100 nm). (b) TEM image of nCOF (isolated state) (scale bar: 100 nm). (c) SEM image of bCOF (scale bar: 1 µm). (d) DLS curves of nCOF and bCOF aqueous solutions. (e) Digital photograph of PAAm‐nCOF‐3 precursor after 5 min sonication with optical microscope image on the right side (scale bar: 200 µm). (f) Digital photograph of PAAm‐bCOF precursor after 5 h sonication with optical microscope image on the right side (scale bar: 180 µm). (g) UV–vis transmittance spectra of precursors of PAAm, PAAm‐bCOF, and PAAm‐nCOF‐3, with inserted digital photographs of the corresponding solutions in glass vials and the resultant hydrogels. (h) Photographs of hydrogels obtained with high resolution. From top to bottom, the samples correspond to PAAm, PAAm‐nCOF‐3, and PAAm‐bCOF, respectively, each with a thickness of 2 mm.

We incorporated nanoscale TFP‐BpyD COF (referred to as nCOF in this work), which was synthesized following previous work [36] (Figure 1b), and bulk COF (referred to as bCOF) [40, 41], which was synthesized using the same monomers for comparison, into in situ polymerizations. The formation of COF structures, as well as the crystallinity and porosity of COFs, are illustrated in Figures S1–S4. Polar groups of the COFs (e.g., N‐H, = N‐, and C = O) provide abundant active sites in COF nanochannels for hydrogen bonding, which promotes the rapid diffusion of monomers into the COF nanochannels. The pore sizes of both COFs are around 1.8 nm, providing sufficient space for the monomers and/or polymer chains to enter the pore channels. nCOF exhibits a nanoscale, fiber‐like structure (Figures 2a,b and Figure S5), while bCOF appears as micro‐scale bulk particles (Figure 2c). In addition, dynamic light scattering (DLS) analysis (Figure 2d) reveals that the diameter of nCOF ranges between 20 and 50 nm. The atomic force microscope (AFM) (Figure S6) suggests nCOF has about 5 nm of thickness. In contrast, bCOF particles are significantly larger, with sizes ranging from 1.2 to 1.5 µm.

nCOF and bCOF reinforced hydrogels were labeled as PAAm‐nCOF‐x (x = 1, 2, 3, 4, and 5, referring to 0.01–0.05 wt.% nCOF with 0.01 wt.% stepping) and PAAm‐bCOF (0.03 wt.% bCOF), respectively. Pure hydrogel without COF was labeled as PAAm. PAAm‐bCOF (0.03 wt.% bCOF) was chosen as the benchmark to provide an optimal balance between loading efficiency and material stability for a reliable comparison. We found that nCOF can be homogeneously dispersed in aqueous pre‐solution after just 5 min of sonication with no particles visible under the optical microscope (Figure 2e). While for bCOF, particles with varying sizes (micro‐scale) were observed randomly dispersed in solutions even after 5 h of sonication (Figure 2f). Furthermore, ultraviolet‐visible (UV–vis) transmittance spectra (Figure 2g) show high transparency (85%–90%) of nCOF solution, while low transparency (10%–20%) is observed for bCOF solution at the wavelength between 650 and 750 nm. That is attributed to the microstructure of bCOF blocking light. After polymerization, the PAAm‐bCOF hydrogel appears cloudier than both the PAAm‐nCOF‐3 and PAAm hydrogels, indicating a good dispersion of nCOF in hydrogels (Figure 2h). The polymerization and formation of hydrogels were confirmed by Fourier‐transform infrared spectroscopy (FTIR, Figure S7). The introduction of COF also improves the thermal stability of the hydrogels by increasing degradation temperature from 200°C (PAAm) to 220°C (PAAm‐bCOF), and to 240°C (PAAm‐nCOF‐x) (Figure S8).

### 2.2 Investigations of Mechanical Properties

**FIGURE 3:**
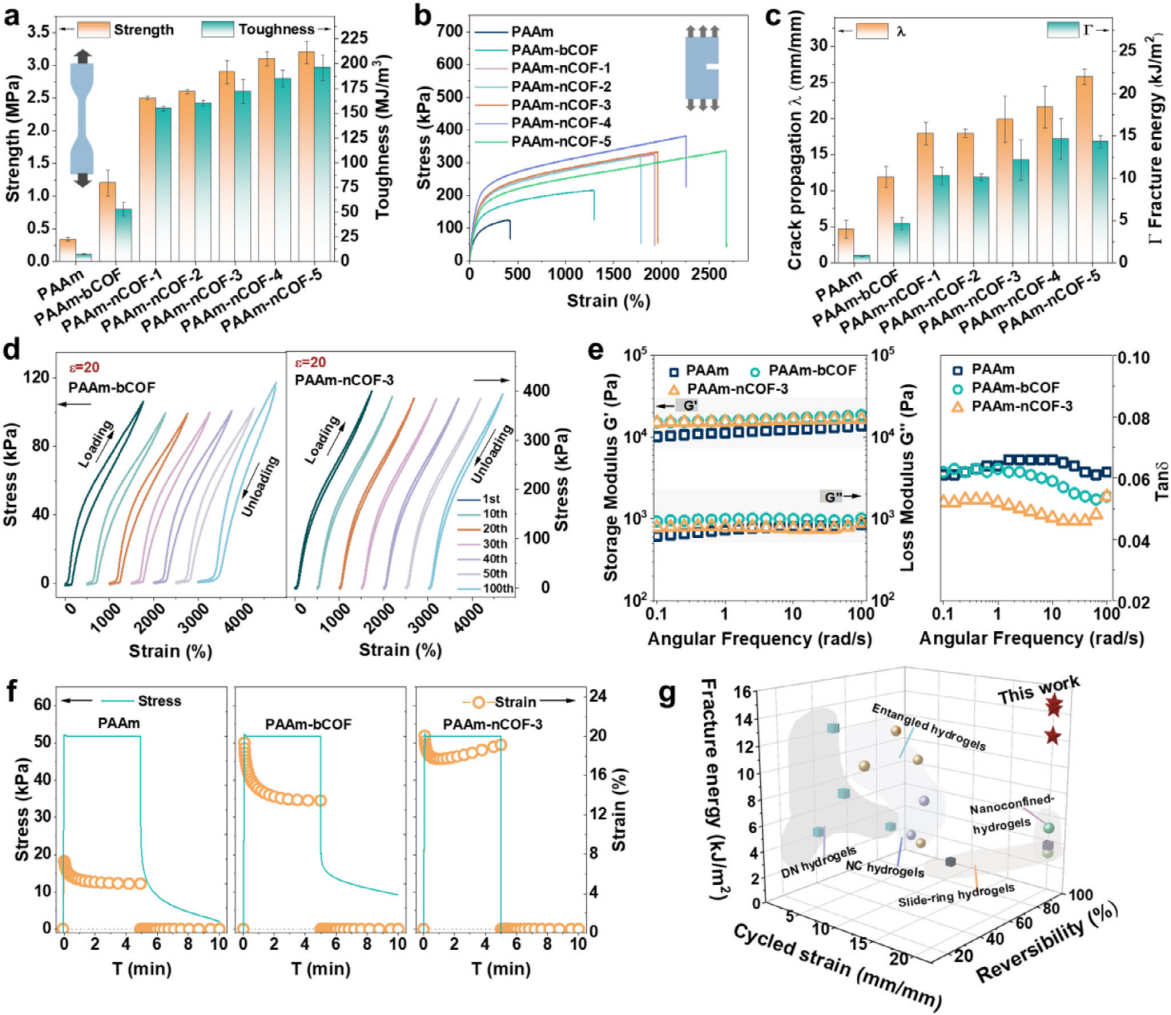
Mechanical and rheological properties of hydrogels. (a) Average strength and toughness of all hydrogels, determined by tensile tests of unnotched samples. (b) Typical stress–strain curves for single‐notched tensile testing of all hydrogels. (c) Average crack propagation strain and fracture energy of all hydrogels, determined by tensile tests of single‐notched samples. (d) Stress–strain curves from the loading‐unloading cyclic test of hydrogel PAAm‐nCOF‐3 and PAAm‐bCOF at a maximum strain of 2000%. From left to right, the horizontally shifted curves respectively represent the 10th, 20th, 30th, 40th, 50th, and 100th cycles. (e) Rheological properties of hydrogels PAAm, PAAm‐bCOF, and PAAm‐nCOF‐3, showing G′, G″, and tanδ as a function of frequency. (f) Stress relaxation curves of hydrogels PAAm, PAAm‐bCOF, and PAAm‐nCOF‐3 with strain consistently controlled at 20% and a recovery time of 10 min. (g) Fracture energy and reversibility with cycled strain (mm/mm) of PAAm‐nCOF‐x compared with other tough hydrogel systems (the listed references are shown in Table S1).

The stress–strain curves (Figure S9) clearly indicate that nCOF significantly reinforced hydrogels. Figure 3a illustrates that with only 0.01 wt.% nCOF loading, the hydrogel obtained an order of magnitude increase for strength from 0.3 to 2.5 MPa, and ∼20 times increase for toughness from 7.5 to 155 MJ/m^3^. With increasing nCOF concentration, the strength and toughness increase and reach the highest value of 3.2 MPa and 196 MJ/m^3^, respectively, belonging to PAAm‐nCOF‐5. Compared with PAAm‐bCOF, hydrogels PAAm‐nCOF‐x exhibit over threefold enhancements in both strength and toughness, underscoring the superior toughening ability of nCOF due to its efficient use of COF nanochannels.

For single‐notched samples (Figures 3b,c and Figure S10), hydrogels PAAm‐nCOF‐x exhibit significantly improved tear resistance compared to both PAAm‐bCOF and PAAm, indicating that abundant physical crosslinking can efficiently eliminate stress concentration at the crack tip, avoiding crack propagation. For example, PAAm‐nCOF‐3 exhibits braking stress, crack propagation strain, and fracture energy of 413 kPa, 2000%, and 12.2 kJ/m^2^, respectively, while for PAAm, the braking stress, crack propagation strain, and fracture energy are only 130 kPa, 465%, and 0.8 kJ/m^2^, respectively. The braking stress, crack propagation strain, and fracture energy of PAAm‐nCOF‐3 are ∼3, ∼4, and ∼15 times higher than those of PAAm, and are ∼2, ∼2, and ∼3 times higher than those of PAAm‐bCOF at the same COF loading.

Cyclic loading‐unloading tensile tests reveal that hydrogels PAAm‐nCOF‐x have hyperelasticity and unexceptional fatigue resistance, which PAAm‐bCOF and PAAm lack. PAAm‐nCOF‐3 exhibited exceptionally low mechanical hysteresis, with ∼93% energy recovery at a 2000% strain (Figure 3d). Even after 100 cycles, it maintained minimal stress relaxation, with only 7% energy dissipation. Similar performance was observed for other nCOF‐reinforced hydrogels (e.g., PAAm‐nCOF‐5, Figure S11). In contrast, PAAm‐bCOF showed large hysteresis loops, exhibiting 16% energy dissipation in the first cycle and 78% energy recovery with increased stress and loop size after 100 cycles. It should be noted that too low an nCOF loading content, such as in PAAm‐nCOF‐1, results in only limited improvement in fatigue resistance compared to the hydrogel control (Figure S12). Moreover, rheology study (Figure 3e and Figure S13) shows that PAAm‐nCOF‐3 has the lowest loss factor (tanδ < 0.05), indicating superior elasticity, whereas PAAm‐bCOF (tanδ = 0.05–0.06) shows only slight improvement over pure PAAm (tanδ = 0.06–0.07).

Stress relaxation, a common cause of mechanical hysteresis, typically reduces energy dissipation over time. Notably, hydrogels PAAm‐nCOF‐x show no stress relaxation but instead demonstrate hyperelasticity and self‐reinforcement under small deformations (20% strain). Figure 3f and Figure S14 indicate that, unlike PAAm and PAAm‐bCOF hydrogels, which exhibit stress relaxation at 20% strain characteristic of typical viscoelastic hydrogels, PAAm‐nCOF‐x hydrogels display increasing stress under constant strain. Upon force release, PAAm‐nCOF‐3 fully recovered instantly, while PAAm took 5 min, and PAAm‐bCOF remained partially deformed. Strain recovery supports this observation, with PAAm‐nCOF‐3 recovering nearly 100%, while PAAm and PAAm‐bCOF recover only 39% and 52.5%, respectively. This phenomenon can be observed under different strains and is repeatable (Figures S15–S17). These results indicate that nCOF templating polymer topological configuration significantly improves polymer elasticity by strong interactions between COF nanochannels and polymer chains or the polymer chains themselves, thereby preventing chain slippages.

High toughness and the ability to withstand consecutive large cyclic deformations are crucial for hydrogel applications, but they can often be improved simultaneously only to a limited extent. We compared the fracture energy‐cyclic deformation‐reversibility relationship of our nCOF‐reinforced hydrogels with other typical hydrogel systems with similar water content (Figure 3g). Traditional hydrogel toughening approaches, such as double‐network (DN) hydrogels, improve toughness but suffer from poor reversibility (30%–85%) and limited cycled deformation (λ = 1–7) [42, 43, 44, 45]. Topological designs improve two of these three factors simultaneously, but not all three. Nanocomposite (NC) hydrogels [21, 46, 47] and entangled hydrogels [8, 14, 15, 48] offer high toughness and high reversibility (∼100%) but limited cyclic deformation (λ < 6), while slide‐ring hydrogels [10, 49] significantly improve the cycled deformation (λ = 20) and reversibility (∼100%) but have low fracture energy (< 5 kJ/m^2^) at high hydroxypropyl‐a‐cyclodextrin (CD) rings loading (18–20 wt.%). In contact, our PAAm‐nCOF‐x hydrogels obtain the highest fracture energy, 14.7 kJ/m^2,^ and very low hysteresis under large deformation (λ = 20). Notably, compared to the current state‐of‐the‐art COF‐reinforced PAAm built by the same matrix structure as ours (crosslinker PEGDA, Mn = 575 g/mol, 0.3 wt.% COF, 1429±32% strain, and 5.4 kJ/m^2^ fracture energy) [28], our PAAm‐nCOF‐x hydrogels nearly double the crack propagation strain and nearly triple the fracture energy using one order of magnitude less nCOF.

### 2.3 Mechanism Study of Toughening

**FIGURE 4:**
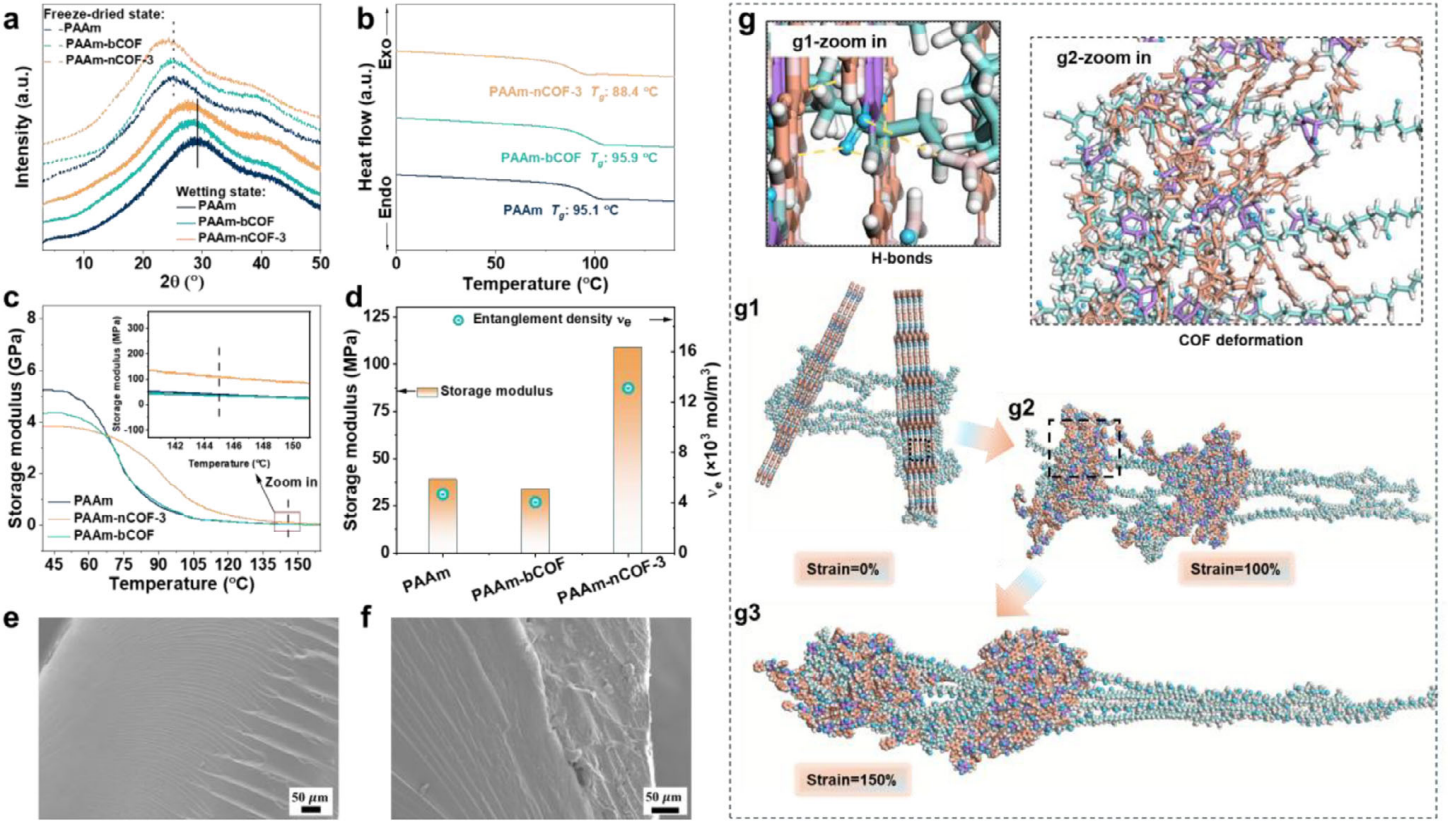
Toughening mechanism studies. (a) XRD patterns of hydrogels PAAm, PAAm‐bCOF, and PAAm‐nCOF‐3 at wetting state and freeze‐dried states. (b) DSC curves of three freeze‐dried polymers. (c) DMA curves of three freeze‐dried polymers. (d) Storage modulus at the rubber platform and entanglement densities of three freeze‐dried polymers. SEM images of the cross‐sections of fractured (e) PAAm‐nCOF‐3 and (f) PAAm‐bCOF (scale bar: 50 µm). (g) Mechanical property simulations of PAAm entangled through threading pore channels of nanoscale COF; g1 to g2 and g3 illustrate how entangled polymer chains pass through the pore channels of nanoscale COF evolve during the stretching process, with no breakage observed; The highest pull‐out energy recorded for this system was 9531.671 kcal/mol.

To investigate the reinforcement mechanism, we examined the structures and fracture surfaces of hydrogels PAAm‐nCOF‐x. X‐ray diffraction (XRD) confirmed an amorphous structure, with all samples displaying a scattering halo centered at about 25° (Figure 4a). Detailed analysis showed that PAAm‐nCOF‐3 hydrogel has a larger van der Waals interaction distance than both PAAm‐bCOF and PAAm, suggesting that the polymer chains in PAAm‐nCOF‐3 likely thread through the pore channels of nCOF, which effectively increases the inter‐polymer chain distances. In contrast, PAAm‐bCOF hydrogel shows a similar distance to that of PAAm, indicating minimal polymer threading. Differential scanning calorimetry (DSC) study (Figure 4b) reveals that the glass transition temperature (*T_g_*) of the PAAm‐nCOF‐3 (88.4°C) is 7°C lower than that of pure hydrogel (95.1°C), suggesting that addition of nCOF effectively weakens the intermolecular interaction of polymer chains due to steric hindrance by threading polymer chains in the nCOF.

Dynamic mechanical analysis (DMA) further confirmed polymer threading. Generally, the storage modulus, G’, in the rubbery state could be used to determine the crosslinking density of polymers. In our hydrogel systems, the covalent crosslinking densities of all hydrogels are the same, as the loading ratio of monomer to crosslinker was kept constant. Also, there were no contributions from polymer crystallization as demonstrated by XRD (Figure S18). As a result, any increase in the G’ due to the addition of COF could be attributed to the increase in polymer chain “entanglement”. G’ in the rubbery state (145°C) was significantly higher for PAAm‐nCOF‐3, reflecting increased entanglement density, *ν_e_*. PAAm‐bCOF has similar G’ and *ν_e_* values to those of PAAm (Figure 4c,d). This result indicates that the hydrogel PAAm‐nCOF‐3 exhibits a significantly higher entanglement density due to the weaving of polymer chains within the nCOF pore channels, resulting in enhanced modulus at high temperatures. For the bCOF‐reinforced hydrogel, the entanglement density is quite similar to that of pure PAAm, suggesting that the threading of polymer chains into bCOF is minimal. Instead, PAAm‐bCOF hydrogels rely on hydrogen bonding with the matrix, i.e., dominated by filler reinforcement [21], rather than facilitating entanglements.

Scanning electron microscopy (SEM) images of the fracture surfaces show that both PAAm‐nCOF‐3 (Figure 4e) and PAAm‐bCOF (Figure 4f) exhibit ductile fractures with visible cracks. PAAm‐bCOF contains irregularly sized holes from microscale bulk COF detachment, whereas PAAm‐nCOF‐3 exhibits an evenly distributed, root‐like pattern, demonstrating superior stress distribution (more SEM images can be found in Figure S19). nCOFs act as rigid, reinforced fillers and physical entanglement points that effectively entangle polymer chains and relieve stress concentration more evenly, leading to significantly enhanced tensile properties and toughness, as discussed above.

Molecular dynamics simulations (Figure 4g and Figures S20–S24) further elucidate the reinforcement mechanism. Figure 4g1 illustrates that hydrogen bonds induce the entanglement of polymer chains by threading them through the pore channels of nanoscale COF. Under constant applied force, pure hydrogel fractures (Figure S14), while nCOF confined hydrogels with chain threading through nanochannels remain undamaged after the deformation and straightening of the polymer chains, accompanied by the deformation of the nCOF (Figures 4g2,g3). In contrast, non‐threading polymer‐nCOF blends exhibit similar failure behavior to pure hydrogels (Figure S23).

Consequently, these results demonstrate that the exceptional mechanical performance of our hydrogels arises from fundamentally different reinforcement mechanisms. In contrast to bCOFs, which exhibit poor dispersibility and fail to effectively induce nanoscale chain entanglement, the rationally designed nCOFs disperse rapidly and homogeneously within the polymerization system. Their abundant and accessible nanochannels generate a pronounced nanoconfinement effect, markedly enhancing nanopore utilization and promoting dense chain entanglement at nanoscale interfaces. As a result, strength, toughness, and fatigue resistance are simultaneously improved.

### 2.4 Application Exploration of nCOF‐PAAm Hydrogel as High‐Performance Electrolytes in Zn‐Ion Batteries

**FIGURE 5:**
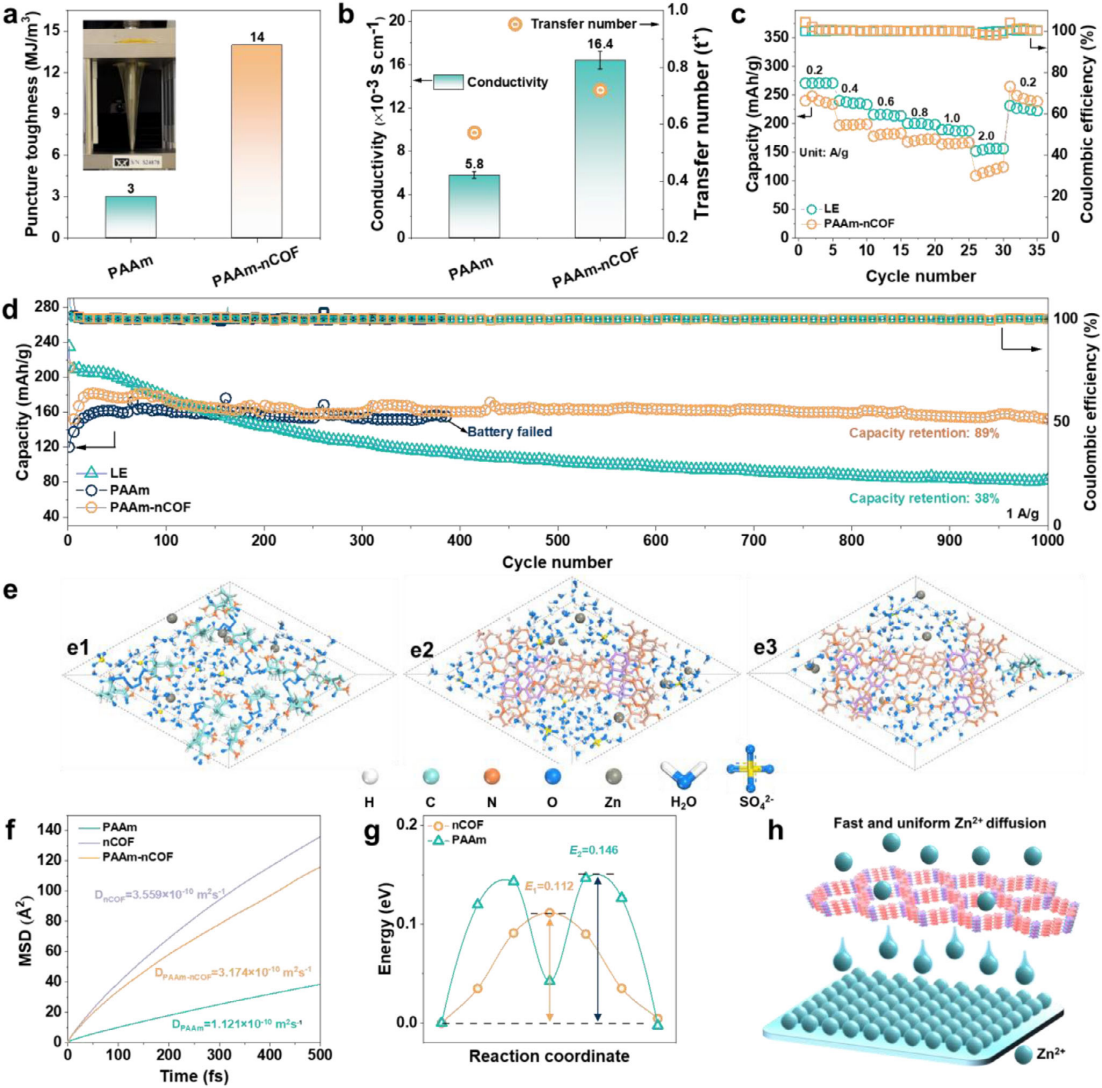
Hydrogel electrolyte performance in Zn‐ion battery. (a) Puncture toughness of hydrogel electrolytes PAAm and PAAm‐nCOF. (Inserted photography of puncture testing of hydrogel electrolyte PAAm‐nCOF.) (b) Ionic conductivity and ion transfer number of hydrogel electrolytes PAAm and PAAm‐nCOF at room temperature. (c) Rate performance of full cells Zn|LE|NVO and Zn|PAAm‐nCOF|NVO. (d) Long cycle performance of full cell Zn||NVO assembled hydrogel electrolyte PAAm‐nCOF at 1 A/g, compared with the batteries assembled with hydrogel electrolyte PAAm and LE. (e) Molecular dynamics simulations of Zn^2+^ diffusion and (f) the diffusion coefficient of Zn^2+^ in three systems: (e1) pure PAAm, (e2) pure nCOF, and (e3) nCOF‐confined PAAm. (g) Migration energy barrier of Zn^2+^ in PAAm and nCOF. (h) Schematic illustration of the role of COF channels in facilitating ion diffusion.

Ionic‐conductive hydrogels PAAm and PAAm‐nCOF were fabricated by incorporating 2 M ZnSO_4_. Puncture resistance tests revealed that incorporating nCOFs significantly enhanced the puncture toughness of hydrogels, increasing it by nearly fivefold compared to the pure hydrogel, from 3 to 14 MJ/m^3^ (Figure 5a), which demonstrates strong potential for resisting dendrite penetration. Furthermore, PAAm‐nCOF exhibited an approximately threefold increase in ionic conductivity compared to PAAm, rising from ∼6 to ∼16 mS·cm^−1^. It also showed an increase in ion transfer number (t^+^) from 0.57 to 0.72 (Figure 5b and Figure S25).

Hydrogel electrolytes for Zn‐ion batteries have been widely reported; however, their performance is often limited by insufficient mechanical robustness and rapid capacity decay [2, 3, 50]. With its synergistic combination of excellent mechanical strength and efficient ion transport, our hydrogel serves as a highly promising quasi‐solid‐state electrolyte material. To further evaluate performance, we synthesized an ammonium vanadium oxide (NH_4_V_4_O_10_, NVO) cathode and employed the ionic‐conductive PAAm‐nCOF hydrogels as electrolytes of the Zn||NVO batteries. For control experiments, a liquid electrolyte (LE), 2 M ZnSO_4_, and an ionic‐conductive PAAm hydrogel were assembled with an anode of Zn and a cathode of NVO. Figure 5c shows that the full cell assembled with the hydrogel electrolyte PAAm‐nCOF exhibits good rate capability, which is significantly enhanced compared to the cell assembled with the LE, due to the reduced water content that inhibits interfacial side reactions.

Typically, a major issue for vanadium‐based cathode materials is their dissolution in the electrolyte during prolonged cycling, which leads to significant performance degradation, especially at low current densities [51, 52]. As shown in Figure 5d, the full cell assembled with the LE retained only 38% of its capacity after 1000 cycles at a current rate of 1 A/g. The dissolution of active material in the electrolyte mainly caused performance degradation. In contrast, the full cell using the hydrogel electrolyte PAAm‐nCOF exhibited excellent electrochemical stability at the same current rate. Although it showed a lower initial capacity, it stabilized after several activation cycles and retained 89% of its capacity after 1000 cycles (charge–discharge curves are shown in Figure S26). On the other hand, the full cell assembled with the hydrogel electrolyte PAAm displayed a similar initial capacity to that of the Zn|PAAm‐nCOF|NVO full cell but failed before reaching 400 cycles. This battery fails due to a short circuit (including a soft short circuit), caused by uneven ion transfer and deposition, and poor puncture toughness to resist dendrite damage (Figure S27). Notably, this performance trend is reproducible across multiple independently assembled cells (Figure S28). SEM images (Figure S29) of anode electrodes from the three full cells after long‐cycle testing show that the anode surface assembled with the hydrogel electrolyte PAAm‐nCOF is flatter compared to those assembled with the other two types of electrolytes, indicating that the hydrogel electrolyte PAAm‐nCOF promotes uniform Zn^2+^ plating, controlled crystal growth, and effective inhibition of dendrite formation.

To elucidate the Zn^2+^ transport behavior in PAAm‐nCOF electrolyte, diffusion coefficient and migration energy barriers of Zn^2+^ in the PAAm system with/without nCOF were calculated. Figures 5e–g illustrate that the incorporation of nCOF into PAAm leads to significantly higher diffusion coefficient and lower migration energy barrier. Consequently, the presence of nCOF not only facilitates faster Zn^2+^ mobility but also bypasses the high‐barrier paths in PAAm, effectively reducing the average migration barrier and enhancing the overall ionic conductivity (Figure 5h) [53, 54]. Moreover, numerous studies have established that COFs possess highly ordered open channels that enable rapid transport of bulky ions [55]. Efficient ion transport through the nCOF network also promotes a more uniform Zn^2+^ flux distribution, mitigates local ion accumulation, and reduces the risk of Zn dendrite formation, thereby enhancing the cycling stability and safety of Zn metal anodes. In comparison, the PAAm hydrogel electrolyte lacks sufficient ionic conductivity and well‐defined nanochannels for ion transport, and its poor puncture resistance likely contributed to premature short‐circuit failure.

Compared with previously reported COF‐based hydrogel systems, our nCOF‐reinforced hydrogel achieves ultratough mechanical properties through nanoconfined polymerization templated by nanoscale COF. The resulting electrolyte simultaneously delivers high ionic conductivity and robust structural stability, enabling superior capacity retention in vanadium‐based full cells, particularly under low current density conditions (Table S2).

## 3 Conclusions

In summary, we present a nanoconfinement‐enabled strategy for fabricating ultratough, multifunctional hydrogels by integrating fully delaminated nCOFs via in situ polymerization. The highly accessible nanochannels and polar functionalities of nCOFs promote directional polymer threading and dense entanglement formation, resulting in single‐network hydrogels that exhibit simultaneously high strength, extreme toughness, hyperelasticity, and minimal hysteresis. Comparative studies with bCOFs reveal the unique advantages of nCOFs in terms of dispersion, interfacial interaction, and stress transfer, enabling a ∼4 fold increase in toughness and a 15 fold increase in fracture energy. Furthermore, the highly improved mechanical robustness enables the hydrogel to function as a durable and dendrite‐resistant electrolyte for Zn‐ion batteries, exhibiting excellent current rate and long‐cycling performance. This work highlights the transformative potential of nCOF structural confinement for tailoring mechanical and electrochemical responses in soft matter. Looking ahead, the structural tunability and modular chemistry of COFs open new pathways for designing adaptive, multifunctional hydrogels in flexible electronics, artificial muscles, and bio‐integrated energy systems.

## 4 Experimental Section

Experimental details and characterizations performed in this work can be found in the Supporting Information.

### 4.1 Statistical Analysis

Error bars in figures of this work mean ± SD.

## Conflicts of Interest

The authors declare no conflicts of interest.

## Supporting information




**Supporting File**: advs74860‐sup‐0001‐SuppMat.docx.

## Data Availability

The data that support the findings of this study are available from the corresponding author upon reasonable request.
