# Supplementary material for: Nanoscale Covalent Organic Framework Confinement Enables Ultratough and Hyperelastic Hydrogels Applied as High‐Performance Electrolytes
Source: Adv Sci (Weinh). 2026 Mar 19;13(30):e22523. doi: 10.1002/advs.202522523 (PMC13248774; doi:10.1002/advs.202522523)
Supplement: Supplementary file 1 — Supporting File: advs74860‐sup‐0001‐SuppMat.docx. [file ADVS-13-e22523-s001.docx]

**Supporting Information**

**Nanoscale Covalent Organic Framework Confinement Enables Ultratough and Hyperelastic Hydrogels Applied as High-Performance Electrolytes**

*Peiyao Yan^†^, Wei Zhao^†^, Hao Wang^†^, Lu Wu^†^, Yedong Ma, Binting Huang, Hantao Xu, Xueyan Liu, Siqi Liu, Xinyu Dong, Xiaoyang Zhang, Wei Zhai, Weiwei Zhang, Lin Xu^*^, Dan Zhao^*^, Chaobin He^*^*

P. Yan, H. Wang, Y. Ma, B. Huang, H. Xu, S. Liu, X. Zhang, C. He

Department of Materials Science and Engineering, National University of Singapore, 9 Engineering Drive 1, 117575 Singapore
E-mail: [msehc@nus.edu.sg](mailto:msehc@nus.edu.sg)

W. Zhao, D. Zhao
Department of Chemical and Biomolecular Engineering, National University of Singapore, 4 Engineering Drive 4, 117585 Singapore

E-mail: [msehc@nus.edu.sg](mailto:msehc@nus.edu.sg)

L. Wu, L. Xu

State Key Laboratory of Advanced Technology for Materials Synthesis and Processing, School of Materials Science and Engineering, Wuhan University of Technology, Wuhan 430070, China

E-mail: [linxu@whut.edu.cn](mailto:linxu@whut.edu.cn)

X. Liu, W. Zhang

Key Laboratory for Advanced Materials and Institute of Fine Chemicals, School of Chemistry and Molecular Engineering, East China University of Science and Technology, Shanghai, China.

X. Dong, W. Zhai

Department of Mechanical Engineering, National University of Singapore, 9 Engineering Drive 1, Singapore 117575, Singapore

C. He

Institute for Materials Research and Engineering (IMRE), Agency for Science, Technology and Research (A∗STAR), 2 Fusionopolis Way, 08-03 Innovis, 138634, Singapore.

†These authors contributed equally; **^*^**Corresponding authors

## Materials

1,3,5-Triformylphloroglucinol (TFP) was obtained from ET Co., Ltd. Acetic acid was obtained from Tokyo Chemical Industry Co., Ltd. [2,2’-Bipyridine]-5,5’-diamine (BpyD) was obtained from BLD Pharm Ltd. Hexadecyl trimethyl ammonium bromide (CTAB), sodium dodecyl sulfate (SDS), dimethyl sulfoxide (DMSO), acetic acid, acrylamide (AAm), poly(ethylene glycol) diacrylate (PEG DA) (Mn = 575 g/mol), 1-hydroxycyclohexyl phenyl ketone (HCPK), and zinc sulfate heptahydrate (ZnSO_4_·7H_2_O) were purchased from Sigma-Aldrich Ltd. Polytetrafluoroethylene (PTFE, battery grade) and Ketjen black (battery grade) were purchased from Sinopharm Chemical Reagent Co., Ltd. Ammonium metavanadate and oxalic acid dihydrate were purchased from Shanghai Macklin Biochemical Co., Ltd. All chemicals were used as received without further purification.

#

# Experimental methods

## Synthesis of nanoscale COF (nCOF)

1,3,5-Triformylphloroglucinol (TFP) (21 mg, 0.1 mmol) was dissolved in 1 mL of dimethyl sulfoxide (DMSO). This TFP solution was added dropwise to a flask containing 58 mL of 0.05M hexadecyl trimethyl ammonium bromide (CTAB) aqueous solution. After ultrasonication, 1.8 mL of 0.05M sodium dodecyl sulfate (SDS) aqueous solution was added to form solution A. Separately, [2,2’-bipyridine]-5,5’-diamine (BpyD) (28 mg, 0.15 mmol) was dissolved in 0.5 mL of DMSO. The solution was added dropwise to a flask containing 58 mL of 0.05M CTAB aqueous solution. After ultrasonication, 1.8 mL of 0.05M SDS solution was added to form solution B. Finally, solutions A and B were mixed, and 5.8 mL of acetic acid was added. After reacting at room temperature for 72 h, an orange colloidal solution was formed. The ideal concentration of TFP-BpyD nano-COF was 0.343 mg·mL^-1^, assuming complete monomer conversion.


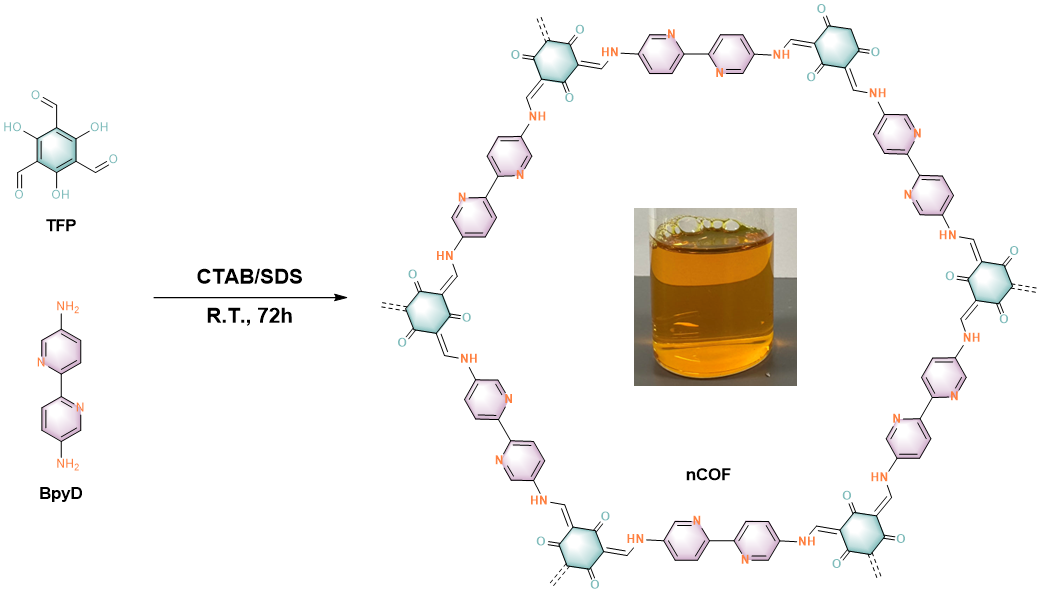


**Scheme S1.** Synthesis route of nCOF.

## Synthesis of bulk COF (bCOF)

The TFP (52.5 mg, 0.25 mmol), BpyD (69.8 mg, 0.0375 mmol), and 12M acetic acid (10 mL) were added into a 20 mL vial and sonicated (Branson Sonifier SFX250 cell disrupter with O.D. 3 mm microtip) in continuous mode for 60min. The resulting solid was washed in sequence with acetone and methanol, followed by a 24 h Soxhlet extraction using THF. The sample was then dried by supercritical carbon dioxide (CO_2_) drying. Finally, the powder was obtained by drying at 80 °C under dynamic vacuum for 12 h.


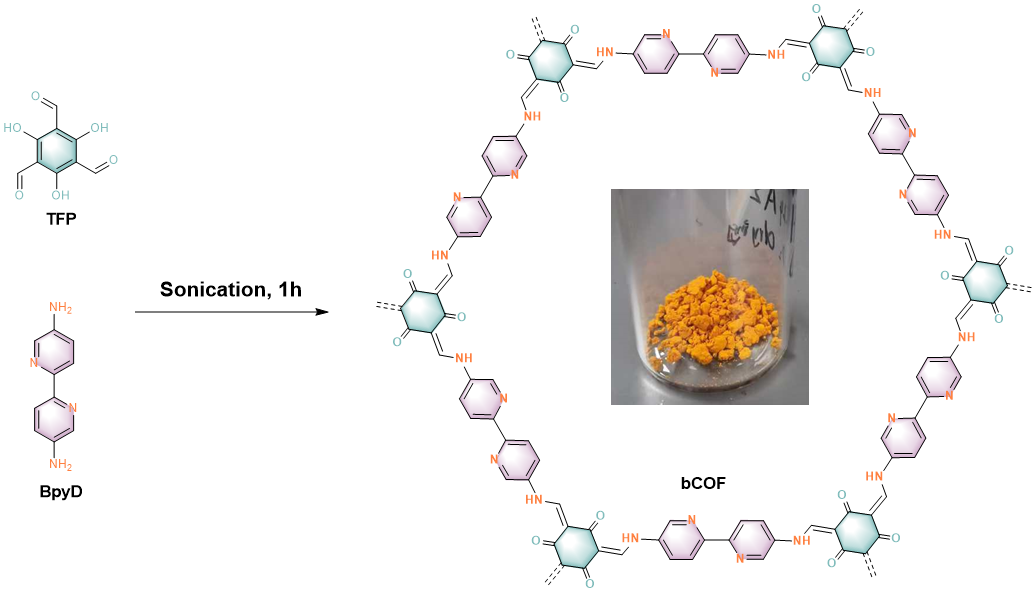


**Scheme S2.** Synthesis route of bCOF.

## Fabrications of hydrogels

***Fabrication of PAAm hydrogel***

AAm was dissolved in deionized water (33 wt.% AAm in the solution), followed by the addition of the photoinitiator HCPK (0.5 wt.% of AAm) and the chemical crosslinker PEG DA (Mn = 575 g/mol, 0.1 wt.% of AAm). The mixture was sonicated for 10 minutes, and the PAAm hydrogel was formed via UV-induced radical polymerization (UV source: 12 J/cm^2^) for 15 minutes at 25 °C.

***Fabrication of PAAm-nCOF-x hydrogels***

AAm was dissolved in deionized water (33 wt.% AAm in solution), followed by the addition of the photoinitiator HCPK (0.5 wt.% of AAm) and the chemical crosslinker PEG DA (Mn = 575 g/mol, 0.1 wt.% of AAm). Various concentrations of nCOF (0.01, 0.02, 0.03, 0.04, and 0.05 wt.% of AAm) were then added. The mixture was sonicated for 5 minutes, and the PAAm-nCOF-x hydrogels were formed via UV-induced nanoconfined polymerization (UV source: 12 J/cm^2^) for 15 minutes at 25 °C.

***Fabrication of PAAm-bCOF hydrogel***

AAm was dissolved in deionized water (33 wt.% AAm in solution), followed by the addition of the photoinitiator HCPK (0.5 wt.% of AAm) and the chemical crosslinker PEG DA (Mn = 575 g/mol, 0.1 wt.% of AAm). bCOF (0.03 wt.% of AAm) was then added. The mixture was sonicated for 4-5 hours to ensure thorough mixing, and the PAAm-bCOF hydrogel was formed via UV-induced nanoconfined polymerization (UV source: 12 J/cm^2^) for 15 minutes at 25 °C.

## Synthesis of hydrogel electrolytes

AAm was dissolved in 2M ZnSO_4_ solution (22 wt.% AAm in solution), followed by the addition of the photoinitiator HCPK (0.5 wt.% of AAm) and the chemical crosslinker PEG DA (Mn = 575 g/mol, 0.1 wt.% of AAm). PAAm hydrogel electrolyte was formed via UV-induced polymerization (UV source: 12 J/cm^2^) for 15 minutes at 25 °C. For PAAm-nCOF hydrogel electrolyte, 1.0 wt.% nCOF was added to the above precursor. The mixture was sonicated for 5 minutes, and the PAAm-nCOF hydrogel electrolyte was formed via UV-induced nanoconfined polymerization (UV source: 12 J/cm^2^) for 15 minutes at 25 °C. All hydrogel electrolytes were prepared as free-standing films and subsequently punched into circular discs with a diameter of 16 mm. The thickness of the electrolyte films was precisely controlled at 500 µm using a polytetrafluoroethylene (PTFE) mold.

## Synthesis of cathode material (NVO)

NH_4_VO_3_ (1.178 g, 10 mmol) was dissolved in 35 mL of deionized water at 80 °C to form a pale-yellow solution. Subsequently, H_2_C_2_O_4_·2H_2_O (1.8918, 15 mmol) was added, and the mixture was magnetically stirred until it turned dark green. The resulting solution was transferred to a 50 mL Teflon-lined stainless-steel autoclave and maintained at 140 °C for 48 h in an oven. After cooling to room temperature, the precipitate was repeatedly washed with deionized water and subsequently dried at 80 °C to obtain the final product.

## Preparation of cathode electrode

The electrode was prepared by blending NVO (70 wt.%), Ketjen black (20 wt.%), and polytetrafluoroethylene (PTFE) binder (10 wt.%). The mixture was then dispersed evenly in isopropanol, rolled multiple times to form a uniform film, and vacuum-dried for 12 hours. Finally, the resulting film was mounted onto a titanium mesh for use. Mass load of the electrode is ~1.5 mg/cm^2^.

## Structural modeling of COFs

Structural atomistic simulations of the possible framework structures were carried out using Material Studio software. The simulated PXRD patterns were determined by the Reflex module. The starting space group for simulated structures was selected as P1.

## Isolation of nCOF to obtain bulk solid

The nCOF colloid was neutralized with 6.8 mL of concentrated ammonia and 100 mL of ethanol, yielding a yellow solid precipitate. The dispersion was centrifuged for 5 min at 8000 rpm, and the supernatant was removed. The solid was redispersed in 50 mL of ethanol and stirred for 30 min before being centrifuged again. This washing procedure was repeated a total of 5 times, and a highly concentrated dispersion in ethanol was obtained. Finally, the sample was activated by supercritical CO_2_ drying to obtain bulk nCOF powder.

## Characterizations

*Fourier-Transform Infrared (FTIR).* FTIR was carried out using a Vertex 70 Fourier Transform Infrared Spectrometer between 500 and 4000 cm^-1^ for 32 scans under a resolution of 4 cm^-1^. FTIR analysis was conducted by using the ATR attachment. All hydrogels were freeze-dried, and COFs were dried using supercritical carbon dioxide before being characterized.

*Differential Scanning Calorimetry (DSC).* Thermal properties of hydrogels and freeze-dried samples were characterized by DSC (PerkinElmer DSC 6000). The method for wetting hydrogels involved a heat/cool/heat cycle for three cycles at a heating and cooling rate of 10 °C/min, with temperatures ranging from -70 to 90 °C. The method for freeze-dried polymers involved a heat/cool/heat cycle for three cycles at a heating and cooling rate of 10 °C/min, with temperatures ranging from -70 to 150 °C. The secondary heating curves were recorded as shown in this work. Glass transition temperatures of polymers were analyzed using the official software.

*Thermogravimetric Analysis (TGA).* The thermal stability of hydrogels, freeze-dried polymers, and COFs was determined using a TA Instruments STD-600 under nitrogen. Wetting hydrogels and freeze-dried polymers were heated from room temperature to 600 °C at a heating rate of 20 °C/min, and COFs were heated from room temperature to 800 °C at a heating rate of 10 °C/min. Samples (5-10 mg) were placed in alumina crucibles for testing.

*Ultraviolet-visible spectroscopy (UV-vis).* The UV-visible transparency spectra of PAAm, PAAm-nCOF-3, and PAAm-bCOF pre-solutions were measured using an Agilent Cary 60 UV-Vis spectrometer. The PAAm-nCOF-3 solution was prepared for testing after 5 minutes of sonication, while the PAAm-bCOF solution required 4-5 hours of sonication before testing. *Gas sorption analysis.* The surface areas and nitrogen adsorption isotherms of samples (at 77.3 K) were obtained using a 3 Flex volumetric adsorption analyzer. Before analysis, the samples were degassed at 120 °C for 15 h under vacuum (10^-5^ bar). Pore size distributions of COFs were obtained by fitting the density functional theory (DFT) model to the adsorption data.

*Powder X-ray Diffraction (PXRD).* PXRD measurements were performed on a MiniFlex 600.

*Scanning Electron Microscopy (SEM).* The hydrogels were quenched in liquid nitrogen and freeze-dried overnight. The fracturing cross-sections were coated with a thin layer of conductive material (Au) before observation by the Zeiss Supra 40 FE SEM. Full cells after long-cycle testing were disassembled, and anode electrodes were observed using the Zeiss Supra 40 FE SEM.

*Cryo-Transmission Electron Microscopy (Cryo-TEM).* The Cryo-TEM analysis was conducted using an FEI Talos F200X G2 microscope, optimized for liquid samples. Specimen preparation involved depositing a small drop of the FNP sample onto a lacey carbon-coated 300-mesh copper grid. Excess liquid was carefully removed by blotting with filter paper, leaving a thin film of the solution on the grid. The sample was then vitrified in liquid ethane and transferred to the microscope, where it was maintained below -170 °C and protected from atmospheric exposure. Imaging was performed with the microscope set to an acceleration voltage of 200 kV, and high-resolution images were captured under low electron dose conditions using a Ceta 4K × 4K camera.

*Transmission Electron Microscopy (TEM).* TEM images were acquired using a JEOL 2100FCs microscope operated at an accelerating voltage of 200 kV. The samples were prepared by drop-casting sonicated ethanol suspensions of the materials onto a copper grid.

*Optical Microscope.* COF particles dispersion performance was observed using an optical microscope (Nikon SMZ18). PAAm, PAAm-nCOF-3, and PAAm-bCOF pre-solutions were used in this test. The PAAm-nCOF-3 solution was prepared for testing after 5 minutes of sonication, while the PAAm-bCOF solution required 4 hours of sonication before testing.

*Dynamic Light Scattering (DLS) Particle Analysis.* The hydrodynamic size distributions of nCOF and bCOF aqueous solutions were analyzed by a Malvern Zetasizer Pro.

*Atomic force microscope (AFM).* Thickness of isolated nCOF particles were measuresed using a Bruker Dimension ICON. The radius of curvature of the tip: ~5 nm.

*Dynamic Mechanical Analysis (DMA).* Stress relaxation of the hydrogels was tested using TA Instruments Q800 DMA. Samples were prepared as rectangular shapes with the dimensions 25 mm$\text{×}$5.0 mm$\text{×}$1.0 mm (L, W, T). The tests were performed under the stress relaxation mode with controlling the tensile strain at 5%, 10% and 20%, as well as controlling relaxation time for 5 mins and 10 mins and recovery time for 3 mins and 5 mins, accordingly. Stress relaxation behavior dependence was recorded. No special environmental control was applied to any sample, and all DMA tests were conducted under room temperature (25 °C) without any humidity control. Frequency sweep (constant strain) with temperature ramping was performed using TA Instruments Q800 DMA. Rectangular samples were used and were freeze-dried before measurements.

*Rheology characterization.* The storage modulus (G') and loss modulus (G″) of the hydrogels were measured using a rotational rheometer (Anton Paar MCR-302) with a 25 mm flat plate. A frequency sweep was conducted under a force of approximately 1 N, with the angular frequency ranging from 0.1 to 100 rad/s.

*Mechanical properties measurements.* Tensile tests of all hydrogels were performed using a universal testing machine (Instron 5500) with a 100 N load cell. Unnotched samples were prepared into dog-bone shapes according to GB/T 528-92/4. An initial length of 2 mm was stretched to failure at a strain rate of 60 mm/min. The toughness of the hydrogels was determined by integrating the area under the stress-strain curve. The hydrogels were prepared in rectangular shapes with dimensions of 60.0 mm × 5.0 mm × 1.0 mm (L, W, T). A single-edge notch was introduced using a sharp blade, with a depth of 30% of the sample’s width. The maximum strain ($\epsilon_{m}$) was obtained by stretching the sample with initial length of 2.0 mm to failure. The fracture energy was calculated as W($\epsilon_{m}$) × L, where W($\epsilon_{m}$) represents the integrated area under the stress-strain curve of the unnotched sample corresponding to the $\epsilon_{m}$ of the notched sample, and L is the sample’s initial length. The average values and error bars for all mechanical data were obtained from 4-5 repeated tests per sample. Cyclic loading-unloading tests were conducted by stretching the unnotched samples at a strain rate of 500 mm/min to 2000% strain for 100 cycles. It is noted that for all samples, there was approximately a 200% strain, where the specimen was stretched beyond the grips after the first cycle and could not return to the gap between the grips. Therefore, the figures shown in this work represent the curves after removing the remaining 200% strain. The recovery and dissipation were calculated by dividing the energy released during unloading by the energy absorbed during loading. The energy released and absorbed was calculated by integrating the area under stress-strain curves. Equations for calculations are shown as follows: Recovery = $\frac{E_{r}}{E_{a}}\times$ 100%; and Dissipation = $(1-\frac{E_{r}}{E_{a}})\times$ 100%, where $E_{r}$ is energy released during unloading and $E_{a}$ is energy absorbed during loading.

*Puncture testing.* The puncture resistance test was carried out using an Instron Universal Mechanical Property Testing Machine equipped with a Pneumatic Puncture Fixture in accordance with ASTM F1306. The upper puncture probe featured a hemispherical tip with a radius of 1.57 mm (0.0625 inch), tapering from 3 to 12 mm over a 50 mm length. The specimen was clamped using a pneumatic fixture with a 35 mm diameter opening and rubber pads on the clamping surfaces to prevent slippage. The lower and upper fittings were Type Dm and Type Om, respectively. The test was conducted at room temperature with a crosshead speed of 10 mm/min and a load cell capacity of 500 N. Force-displacement curves were recorded during the test, and the peak puncture force was used to evaluate the material's puncture resistance.

*Ionic conductivity measurement.* Electrochemical impedance spectroscopy (EIS) in the frequency range of 1M Hz-1 HZ with a magnitude of 10 mV (at open circuit voltage) was obtained by an electrochemical workstation (Multichannel Electrochemical Analyser, IVIUMnSTAT, NL). The ionic conductivity of hydrogel electrolytes was determined from electrochemical impedance spectra, with the electrolyte sandwiched between two stainless-steel sheets and secured by plastic spacers. The equation used for the calculation of ionic conductivity σ (mS cm^−1^) is $\text{σ=}\frac{\text{L}}{\text{R}_{\text{b}}\text{×S}}\text{×1000}$, where L (cm) is the distance between the two stainless steel sheets, R_b_ (Ω) is the bulk resistance (intercept at Z′ axis), and S is the contact area (cm^2^) between stainless-steel sheets.

*Ion transfer number measurement.* The Zn ion transfer number (t^+^) was obtained using a Zn||Zn symmetric cell by a chronoamperometry test with a DC voltage amplitude of 10 mV. The EIS tests were taken before and after DC polarization. The t^+^ can be calculated by the equation: $\text{t}^{\text{+}}\text{=}\frac{\text{I}_{\text{SS}}\left( \text{△}\text{V-}\text{I}_{\text{0}}\text{R}_{\text{0}} \right)}{\text{I}_{\text{0}}\left( \text{△}\text{V-}\text{I}_{\text{SS}}\text{R}_{\text{SS}} \right)}$, where I_0_ and I_SS_ are the initial and steady-state DC current, respectively; R_0_ and R_SS_ are the initial and steady-state interfacial resistances, respectively.

*Battery test.* The CR2032-type coin cells were assembled using a zinc metal anode and NVO cathode, with three types of electrolytes: glass fiber separator (thickness: 250 $\text{μ}$m) plus 2 M ZnSO_4_ aqueous solution (120 ul), PAAm hydrogel electrolyte, and PAAm-nCOF hydrogel electrolyte. Galvanostatic charge-discharge measurements were performed using a NEWARE battery testing system (NEWARE_BTS_8.0.1, China) within a voltage window of 0.4-1.4 V (vs. Zn^2+^/Zn).

## Computational calculation for mechanical properties

Structural atomistic simulations of the COF structure were carried out using the Material Studio software. Optimization and dynamics calculations were performed using molecular dynamics (MD) simulations in LAMMPS, applying the polymer consistent force field (PCFF). Initially, the system underwent 5000 steps of conjugate energy minimization, followed by a 100 ps simulation under constant temperature (300 K) and constant volume conditions. Subsequently, the system was equilibrated for an additional 100 ps under constant temperature (300 K) and constant pressure (1 bar).

The composite system model was then subjected to stretching, with the thickness of the upper and lower surfaces fixed at 0.1 nm. Zero pressure was maintained in the X, Y, and Z directions for 300 ps. A tensile load was subsequently applied along the Z axis at a strain rate of 2$\times$10^5^ s^-1^. To ensure zero force along the Z axis, NPT integrals and the Nose/Hoover thermostat were used during the stretching simulation. Periodic boundary conditions were applied in all directions, and atomic stresses in the MD simulation were calculated using the stress tensor in LAMMPS.

## Calculations of diffusion coefficient and migration energy barriers

Density functional theory (DFT) calculations were performed using the Vienna Ab initio Simulation Package (VASP)^[1, 2]^, employing the Perdew-Burke-Ernzerhof (PBE) functional within the generalized gradient approximation (GGA) for exchange-correlation interactions^[3]^. Core-electron interactions were described using projector-augmented wave (PAW) pseudopotentials^[4]^, with van der Waals corrections incorporated via the DFT-D3 method^[5]^. The Brillouin zone was sampled using Monkhorst-Pack k-point meshes with a reciprocal space density of 0.04 Å^-1^, while electronic and ionic relaxations were considered converged when total energy changes between successive self-consistent cycles fell below 1×10^-5^ eV and maximum residual forces on any atom were reduced to 0.05 eV/Å. To investigate Zn²⁺ migration mechanisms, a 1×1×2 supercell of a covalent organic framework (COF) was constructed to simulate ion diffusion channels, complemented by optimized polyacrylamide (PAAm) fragments to examine local coordination environments. Transition states along migration pathways were identified using the climbing image nudged elastic band (CI-NEB) method^[6]^, with five intermediate images interpolated between the initial and final states.

Molecular dynamics (MD) simulations were conducted to investigate the diffusion behavior of Zn^2+^ ions in three distinct systems: (1) pure PAAm (PAAm), (2) pure nCOF system (nCOF), and (3) nCOF-confined PAAm composite (PAAm-nCOF). These systems were constructed within an identical simulation cell dimension defined by a 1×1×4 nCOF supercell. For the PAAm-nCOF system, the crystalline nCOF scaffold was retained, with a single extended PAAm polymer chain embedded within its z-aligned interstitial channel, solvated with 125 H_2_O molecules and ionized with 5 Zn^2+^/5 SO_4_^2−^ ions. Conversely, the pure PAAm system utilized only the geometric dimensions of the 1×1×4 supercell without the nCOF scaffold, instead containing multiple periodically arranged PAAm chains (including both single and double chains) oriented parallel to the z-axis, with identical solvation (125 H_2_O) and ionization (5 Zn^2+^/5 SO_4_^2−^). The pure nCOF system retained the crystalline scaffold without polymer incorporation, solvated with 187 H_2_O molecules and ionized with 7 Zn^2+^/7 SO_4_^2−^ ions to maintain equivalent ion concentration (≈ 2 M) across all systems. All systems underwent a multi-step equilibration protocol. Initial structures were subjected to 20 cycles of simulated annealing in the NPT ensemble, alternating between 300 and 500 K (20 ps per temperature) at a constant pressure of 0.0001 GPa (≈ 1 atm). Subsequently, systems were optimized at 298 K and 0.0001 GPa for 20 ps under NPT conditions using the Nosé-Hoover-Langevin (NHL) thermostat^[7]^. Production simulations were then conducted in the NVT ensemble at 298 K for 20,000 ps to sample diffusion trajectories. The COMPASSⅢ force field was employed throughout all simulations^[8]^, with van der Waals interactions modeled using a 12 Å cutoff and long-range electrostatics treated via the Atom-based method. Mean-squared displacement (MSD) analysis of Zn^2+^ ions was performed over the final 500 ps of production trajectories.

# Supporting results

***Supporting notes regarding characterizations of COFs:*** The formation of both keto-enamine COFs was confirmed using Fourier-transform infrared (FTIR) spectra (Figure S1). Both keto-enamine COFs have -CH-NH- linkages, which could serve as active sites to interact with the amine groups of monomers and polymers via hydrogen bonds. Crystallinity and porosity of both COFs were determined using powder X-ray diffraction (PXRD, Figure S2) and 77 K nitrogen (N_2_) gas sorption (Figure S3) measurements. Thermogravimetric analysis (TGA) conducted under a N_2_ atmosphere confirmed the good thermal stability of both COFs, with degradation temperatures observed at 350 °C (Figure S4).


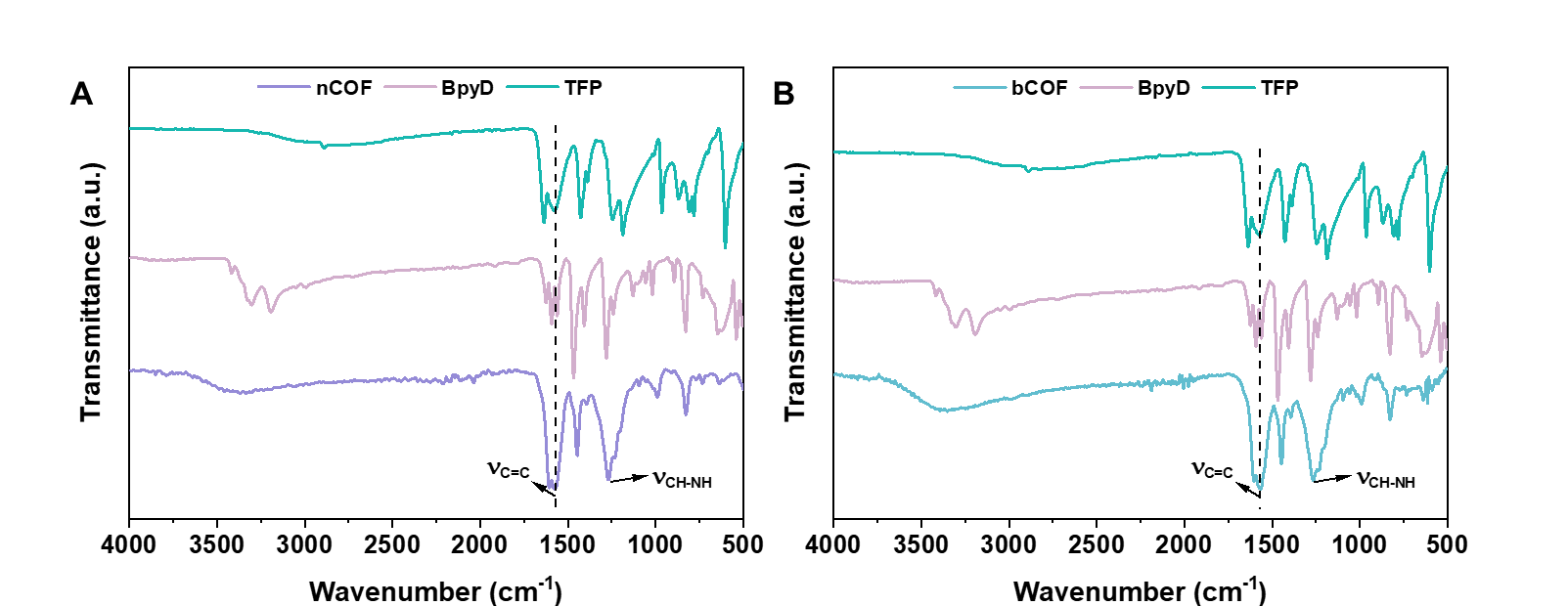


**Figure S1.** (A) FTIR spectra of isolated nCOF and corresponding starting materials. Peaks at around 1570 and 1286 cm^-1^, corresponding to C=C and CH-NH bonds, respectively, indicate that a keto-enamine linkage was formed in nCOF. (B) FTIR spectra of bCOF and corresponding starting materials. Peaks at around 1570 and 1286 cm^-1^, corresponding to C=C and CH-NH bonds, respectively, indicate that a keto-enamine linkage was formed in bCOF.


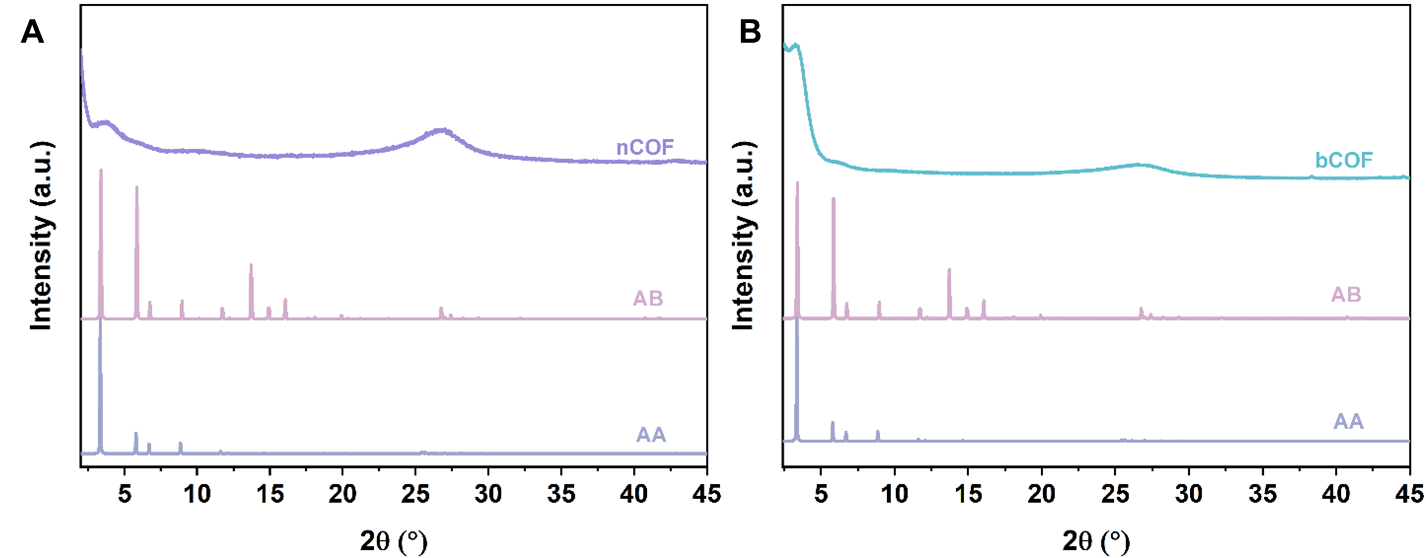


**Figure S2.** (A) PXRD patterns of isolated nCOF compared with the calculated models. The pattern exhibits a relatively strong diffraction peak around 3.4°, corresponding to the (100) reflection. (B) PXRD patterns of bCOF compared with the calculated models. The pattern exhibits a relatively strong diffraction peak around 3.4°, corresponding to the (100) reflection.


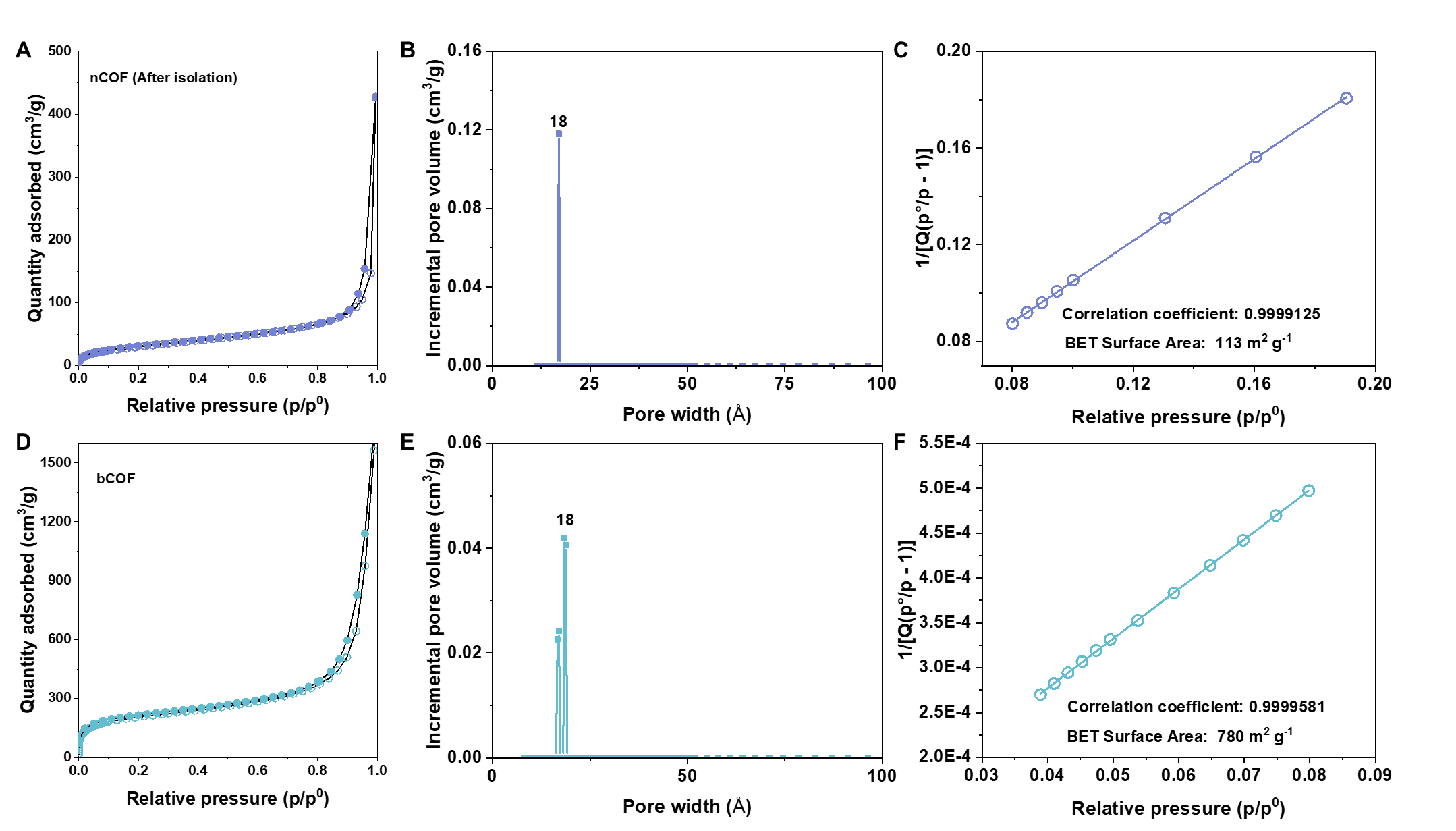


**Figure S3.** N_2_ sorption measurements of isolated nCOF: (A) N_2_ adsorption and desorption profiles at 77.3 K, (B) pore size distribution profile calculated by DFT, (C) BET surface area plot derived from N_2_ sorption isotherm. N_2_ sorption measurements of bCOF: (D) N_2_ adsorption and desorption profiles at 77.3 K, (E) pore size distribution profile calculated by DFT (cylindrical pores model was employed), (F) BET surface area plot derived from N_2_ sorption isotherm.


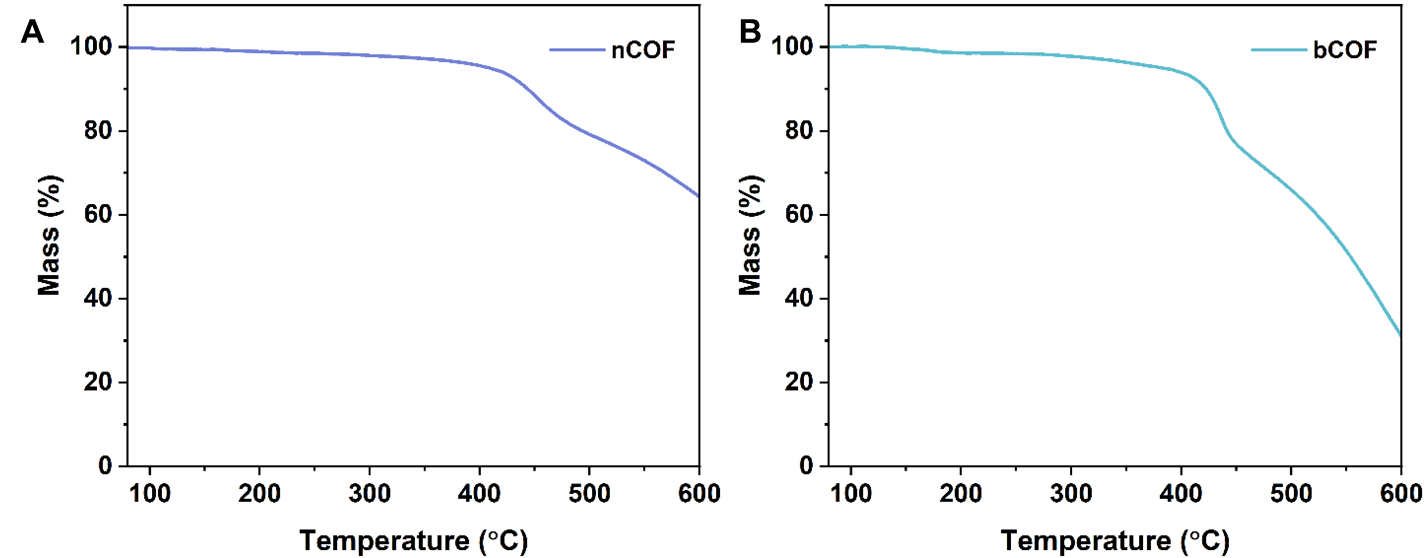


**Figure S4.** TGA curves (under N_2_ atmosphere) of (A) nCOF and (B) bCOF.


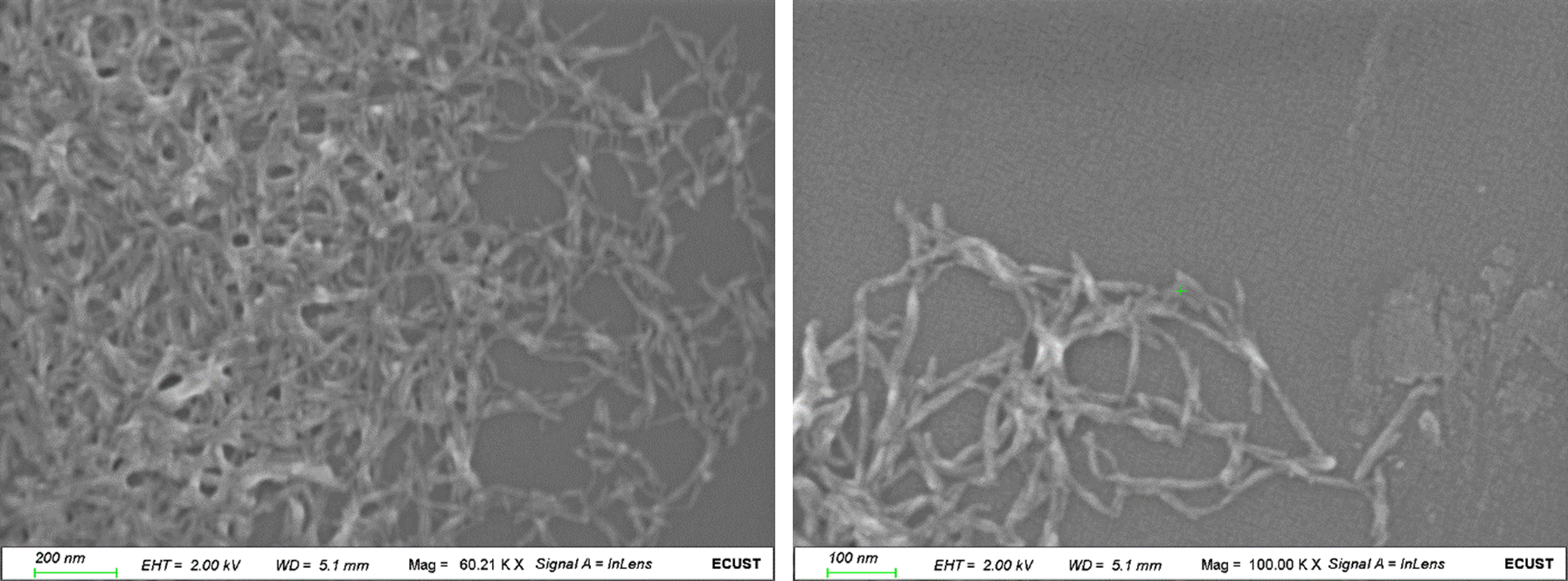


**Figure S5.** SEM image of isolated nCOF.


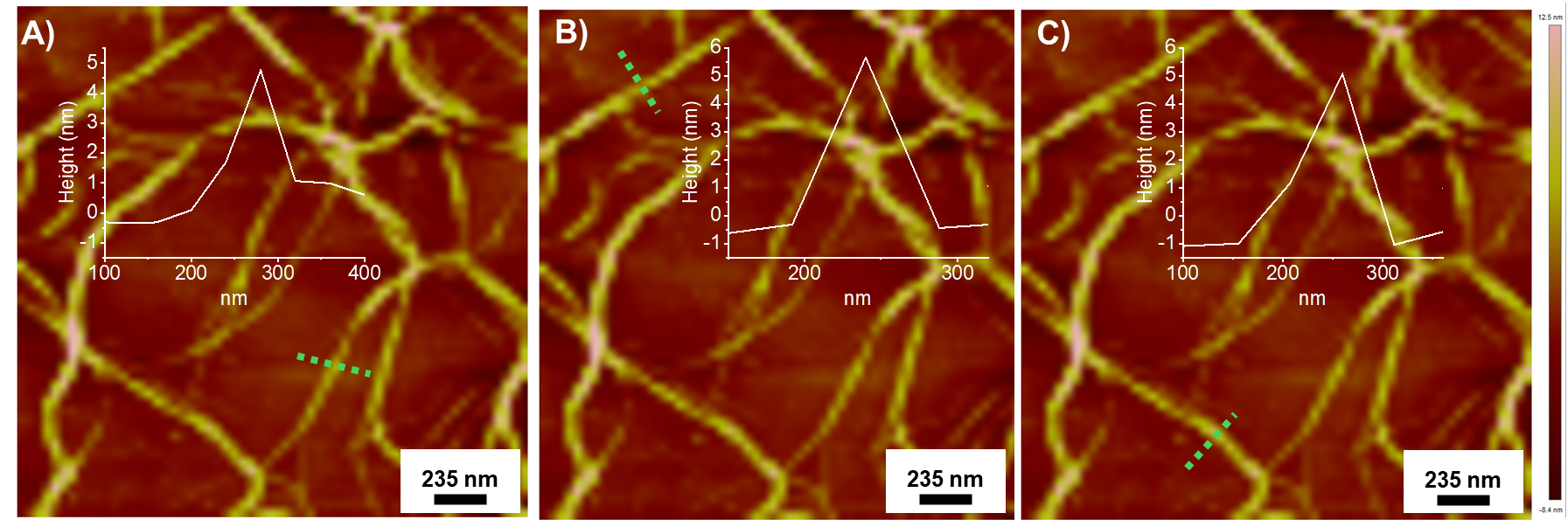


**Figure S6** AFM height images with the corresponding cross-section analysis along the indicated dashed green line. Thickness distribution at multiple positions within a single scan area is presented in panels (A), (B), and (C). The thickness distribution across these sites averages 5 nm.


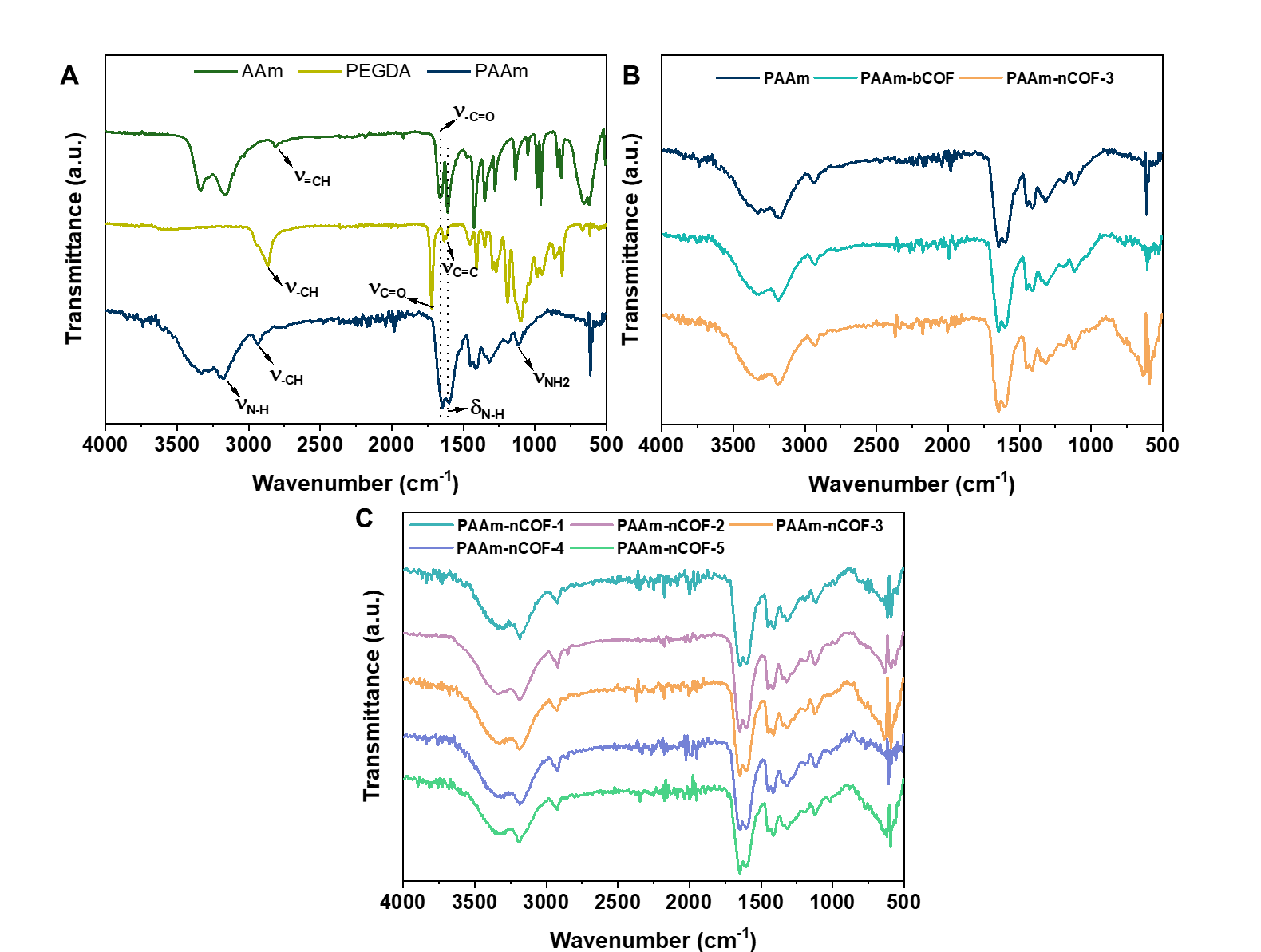


**Figure S7.** FTIR spectra of (A) hydrogel PAAm (after freeze drying) and corresponding starting materials, (B) hydrogels PAAm, PAAm-nCOF-3, and PAAm-bCOF (after freeze drying), and (C) hydrogels PAAm-nCOF-1, PAAm-nCOF-2, PAAm-nCOF-3, PAAm-nCOF-4, and PAAm-nCOF-5 (after freeze drying). The peaks at 2810 and 1628 cm^-1^, attributed to the C=C bonds of the monomers, disappear after polymerization. Typical peaks are at 3327 and 3184 cm^-1^ (attributed to N-H stretching vibration), 1646 cm^-1^ (attributed to C=O stretching), 1602 cm^-1^ (attributed to N-H bending of amide), and 1118 cm^-1^ (corresponding to NH_2_ in-plane bending). That suggests that all characteristic peaks of PAAm appeared. Due to the overlapping of the characteristic peaks of COF and PAAm and the relatively low mass percentage of COF, no distinct COF-related peaks were observed in the FTIR spectra of PAAm-nCOF-x and PAAm-bCOF.

**
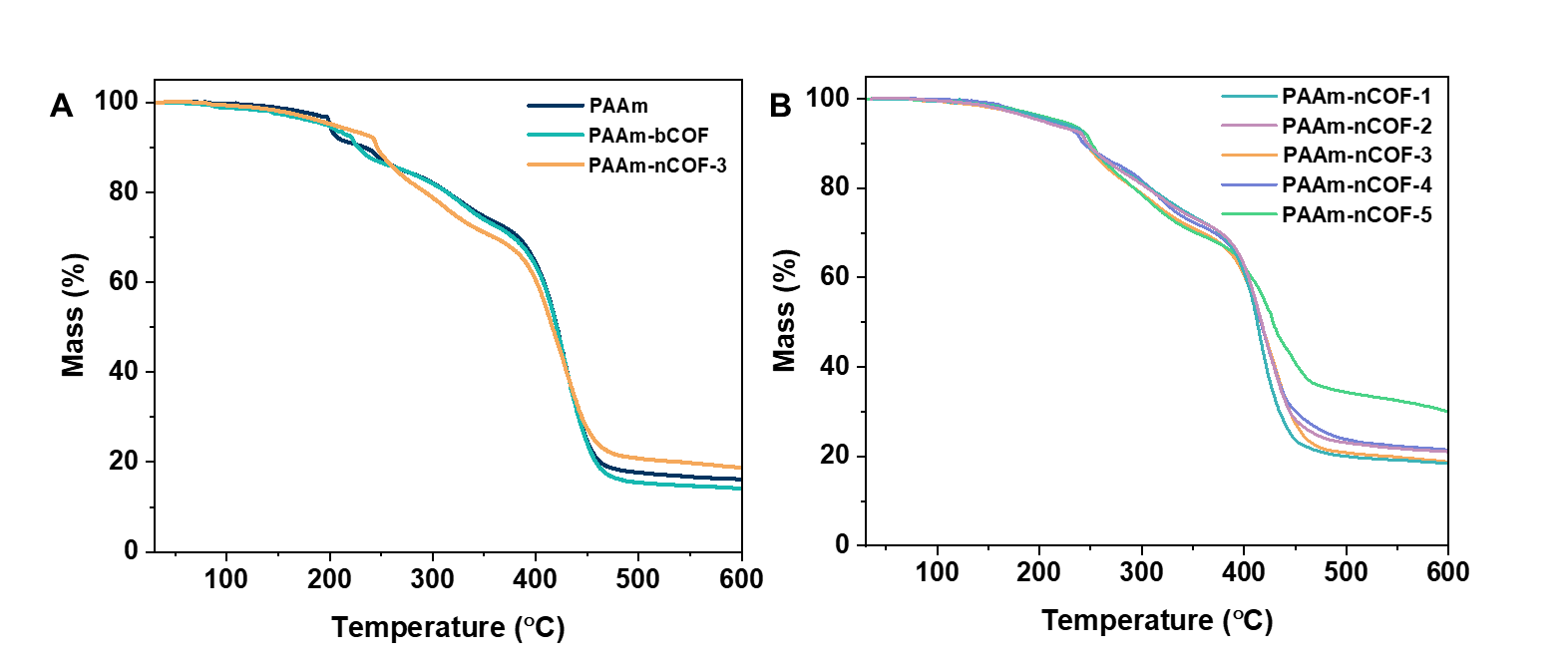
**

**Figure S8.** TGA curves (under N_2_ atmosphere) of hydrogels (A) PAAm, PAAm-bCOF, and PAAm-nCOF-3; (B) PAAm-nCOF-1, PAAm-nCOF-2, PAAm-nCOF-3, PAAm-nCOF-4, and PAAm-nCOF-5 (after freeze drying). The degradation temperature is defined as the temperature at which a 5% weight loss occurs.


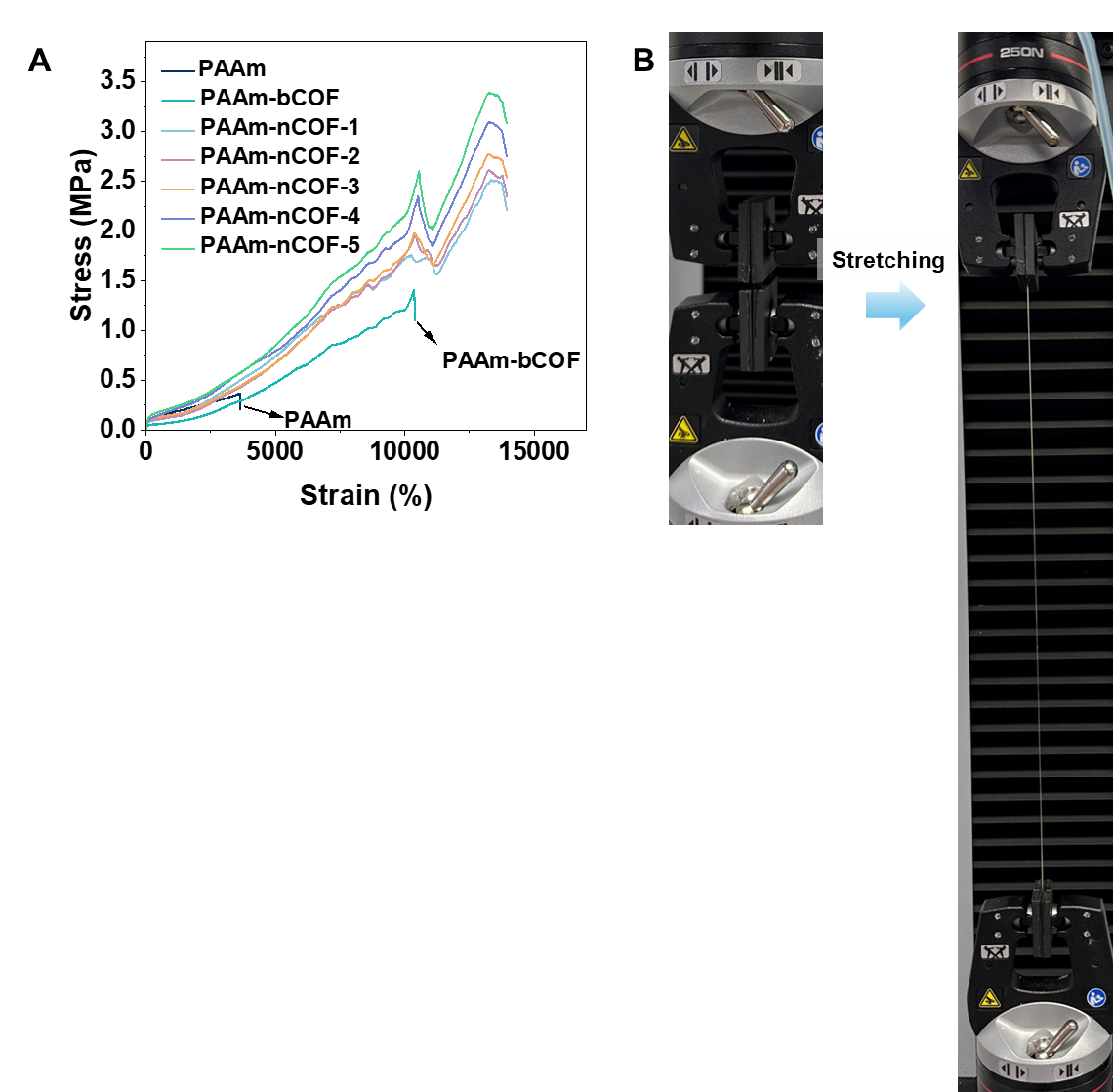


**Figure S9.** (A) Typical stress-strain curves of all hydrogels. (B) Photographs of the PAAm-nCOF-3 hydrogel from the initial state (left) to a strain of approximately 13000% (right).


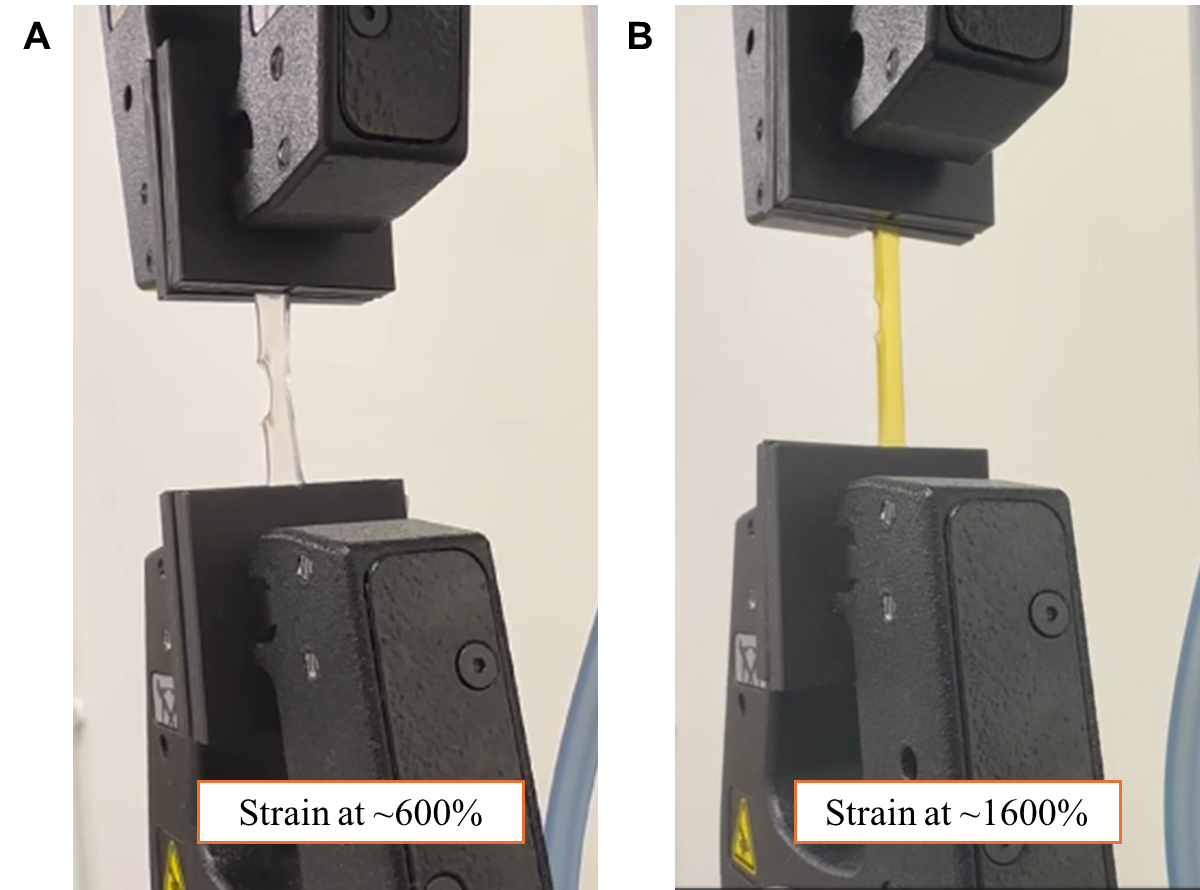


**Figure S10.** Photographs of (A) the notched hydrogel PAAm stretched at an appropriate strain of 600%, and (B) the notched hydrogel PAAm-nCOF-3 stretched at an appropriate strain of 1600%.


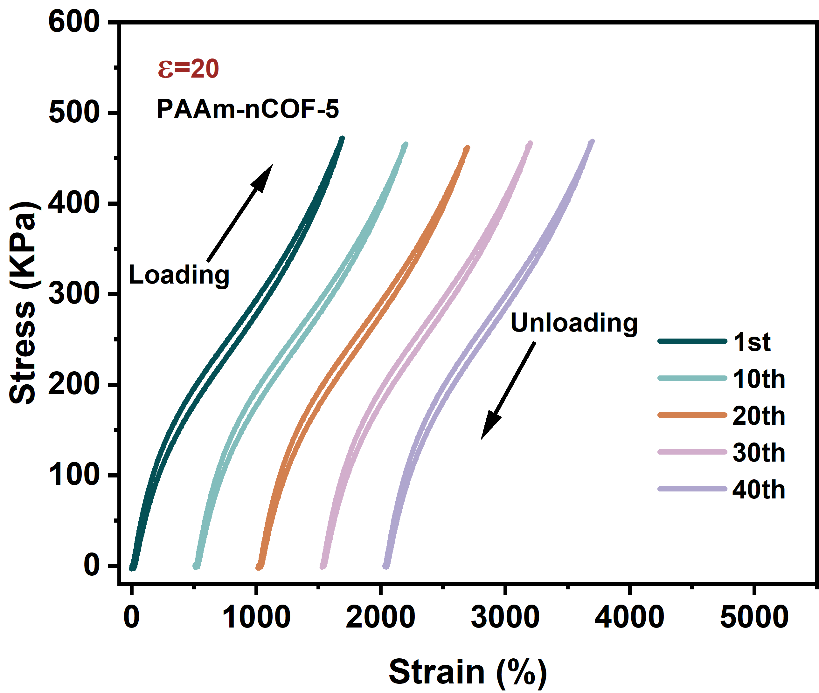


**Figure S11.** Stress-strain curves of the loading-unloading cyclic test of hydrogel PAAm-nCOF-5 with a maximum strain of 2000%. Horizontally shifted curves represent the 10th, 20th, 30th, and 40th cycles.


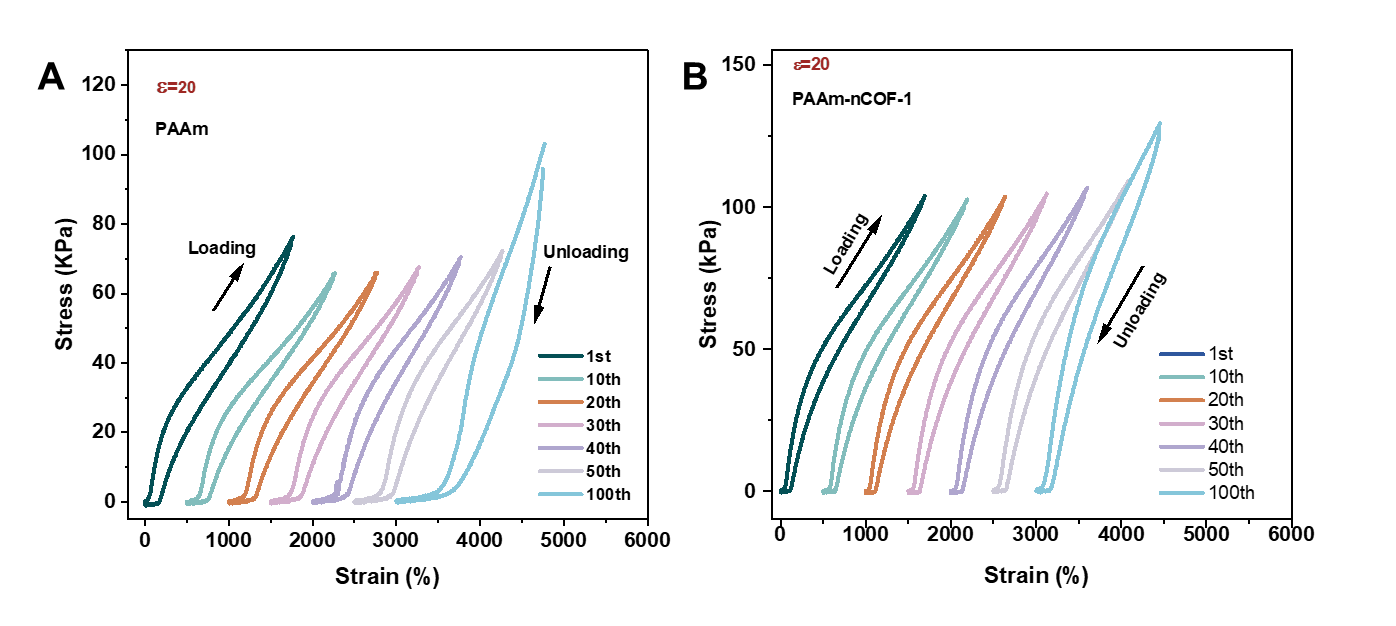


**Figure S12.** Stress-strain curves of the loading-unloading cyclic test of hydrogels PAAm and PAAm-nCOF-1 with a maximum strain of 2000%. Horizontally shifted curves represent the 10th, 20th, 30th, 40th, 50th, and 100th cycles.


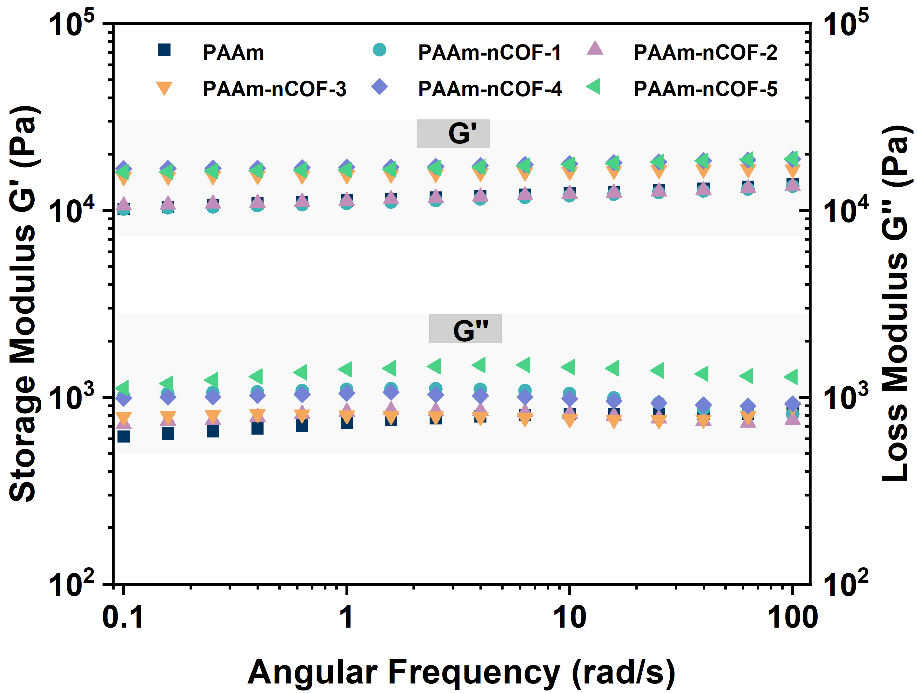


**Figure S13.** The storage modulus (G’) and loss modulus (G’’) of the hydrogels PAAm and PAAm-nCOF-x as functions of frequency.


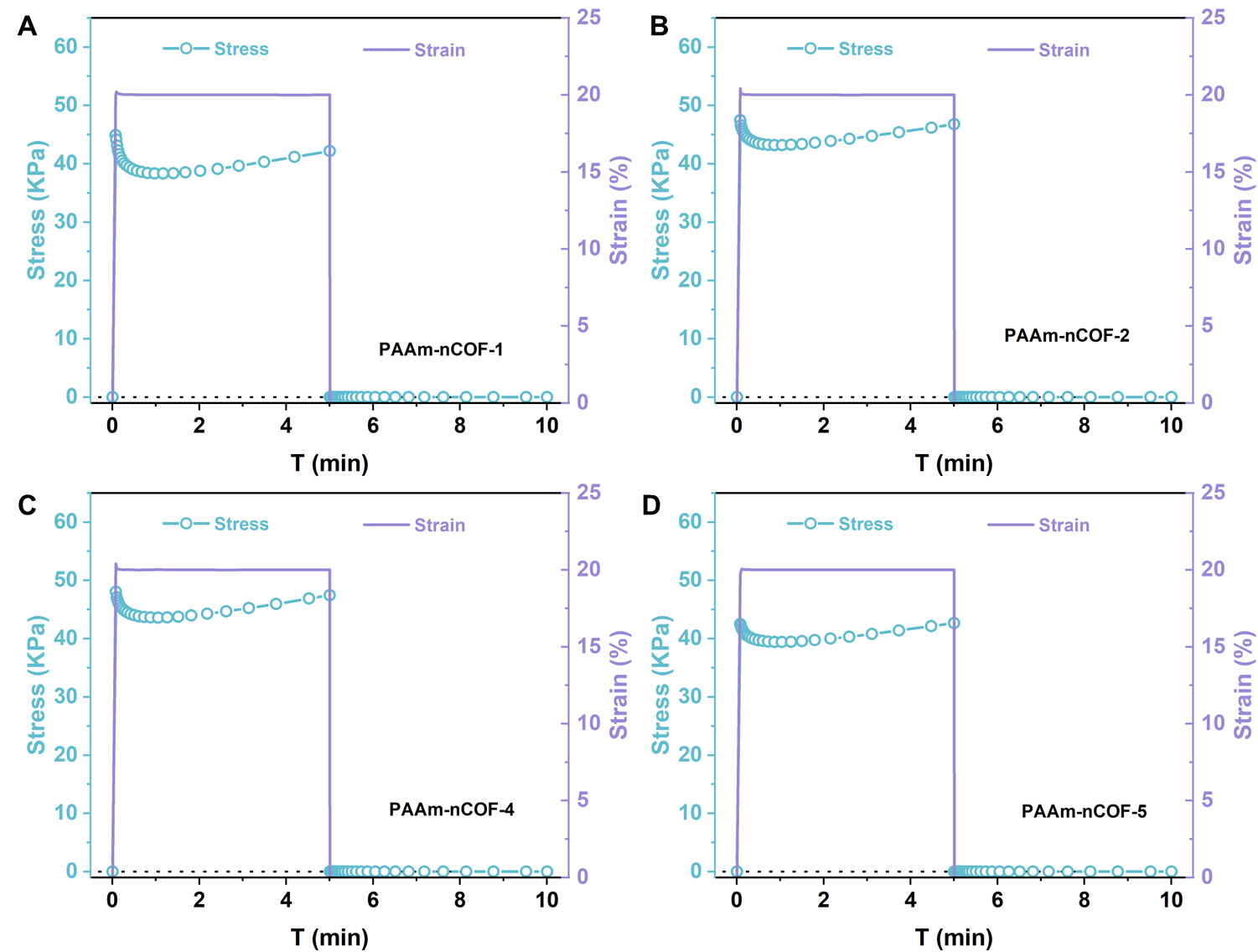


**Figure S14.** Stress relaxation behaviors of hydrogels (A) PAAm-nCOF-1, (B) PAAm-nCOF-2, (C) PAAm-nCOF-4, and (D) PAAm-nCOF-5 with a pre-applied consistent strain of 20%.


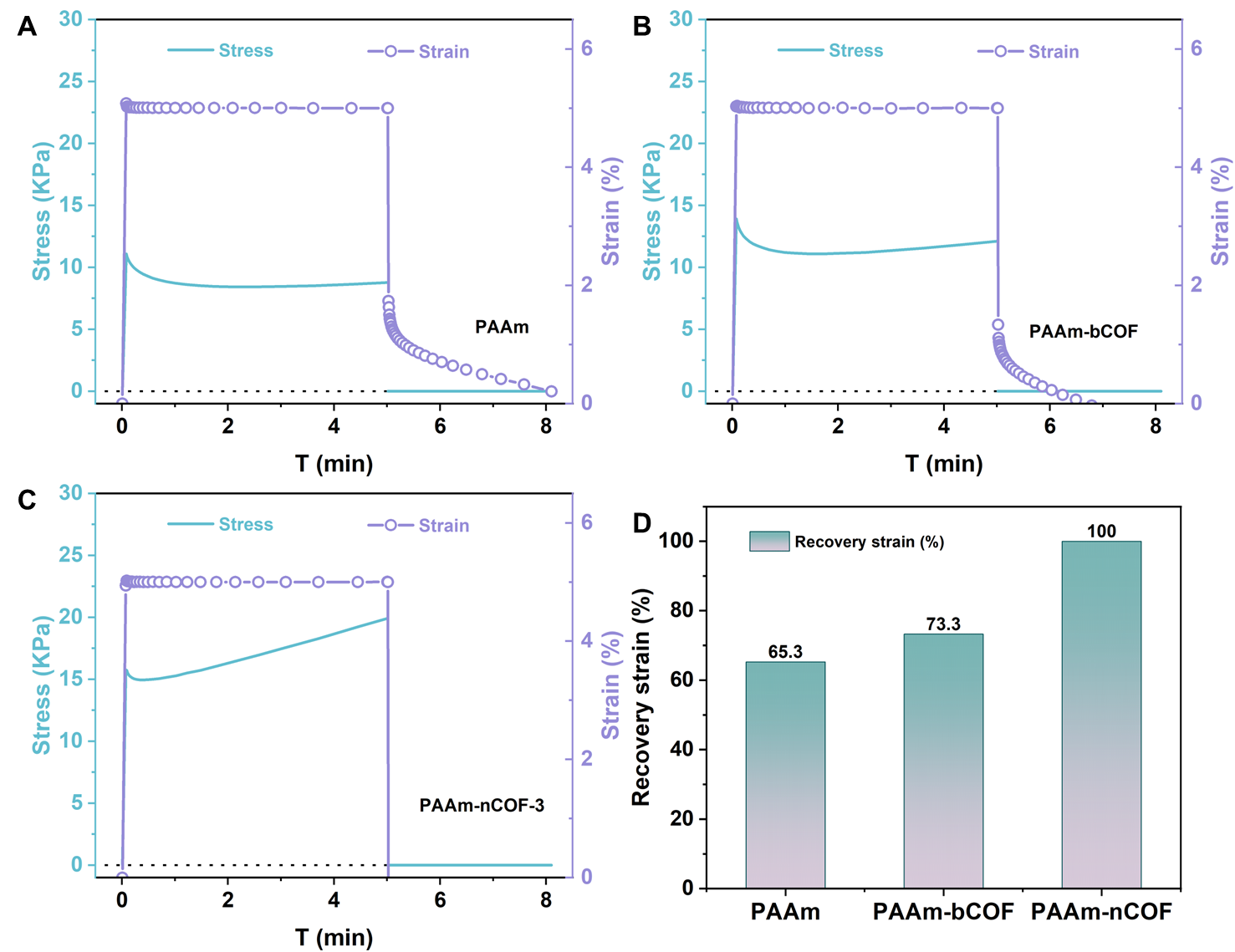


**Figure S15.** Stress relaxation behaviors of hydrogels (A) PAAm (B) PAAm-bCOF (C) PAAm-nCOF-3 with pre-applied consistent strain of 5%. (D) Strain recovery rate immediately after stress release.


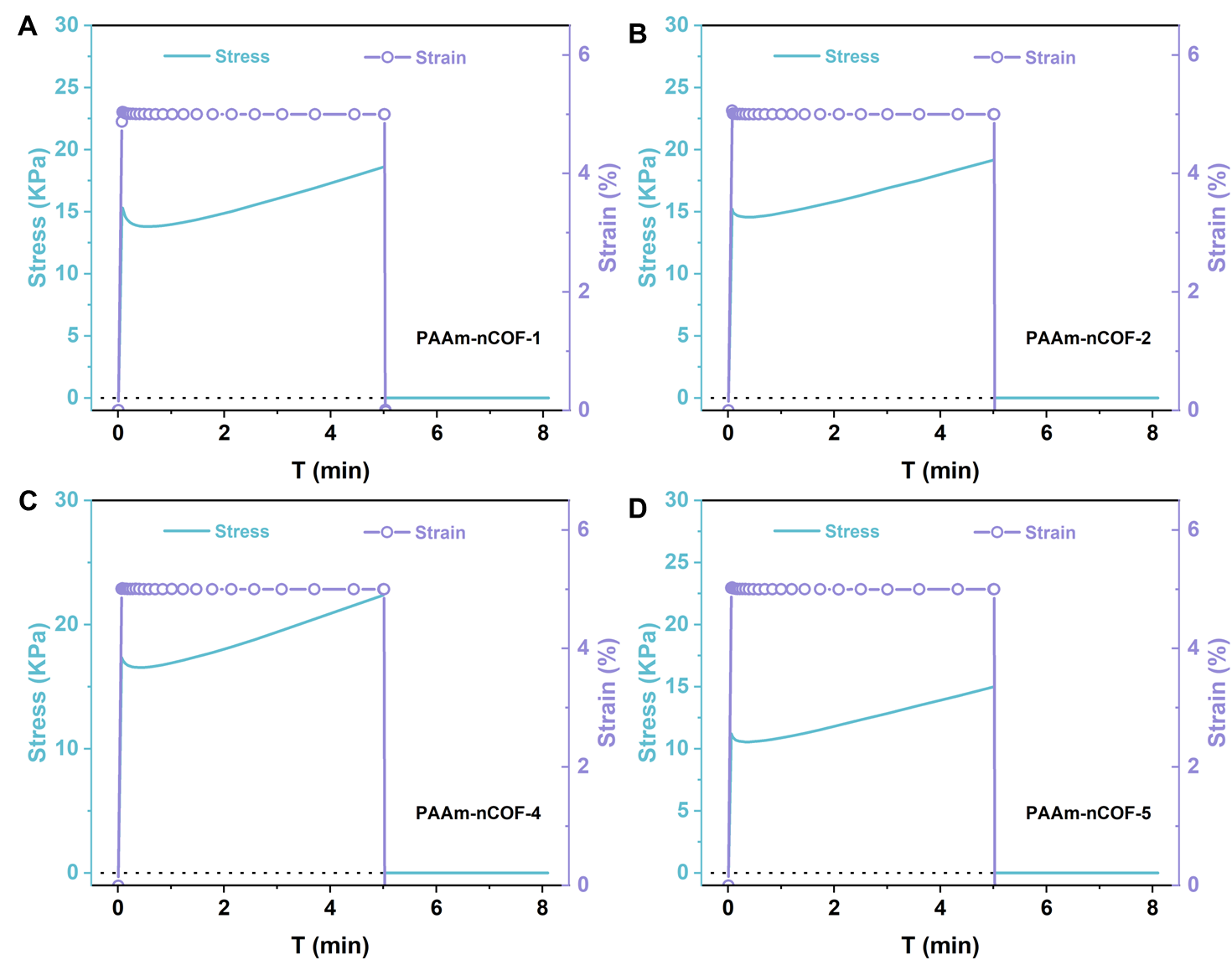


**Figure S16**. Stress relaxation behaviors of hydrogels (A) PAAm-nCOF-1 (B) PAAm-nCOF-2 (C) PAAm-nCOF-4 (D) PAAm-nCOF-5 with pre-applied consistent strain of 5%.


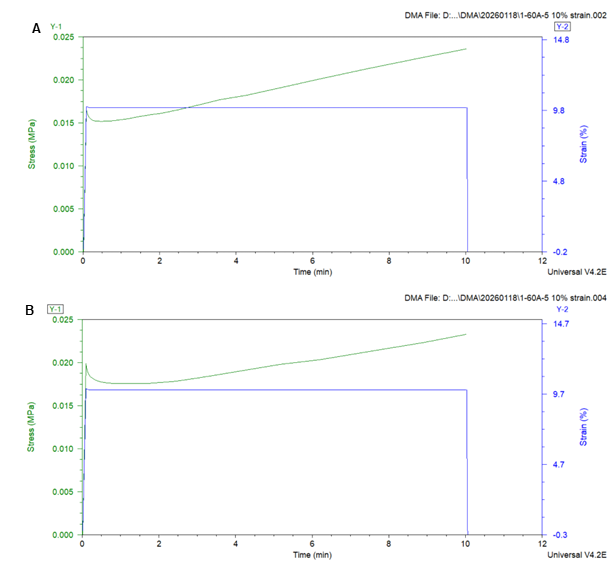


**Figure S17**. Original stress relaxation data for PAAm-nCOF-5, showing replicate curves obtained from two independent specimens (A and B) under a constant pre-applied strain of 10%, demonstrating the reproducibility of the results. The images are screenshots of raw data as displayed in the TA Instruments analysis software.

**Table S1.** Summary of references plotted in Figure 2C.

| Reference number | Citation | Polymer (reinforcement mechanism) | Water content (%) | Fracture energy (J/m^2^) | Ultimate Stress (single-notched) (kPa) | Ultimate Strain (single-notched) (%) | Reversibility (λ_cycle_) |
| --- | --- | --- | --- | --- | --- | --- | --- |
| 10 | Liu et al., Science 372, 1078–1081 (2021) | PEG-CD slide ring gel | 54 | 3600 | ~400 | 250 | ~100% (20) |
| 49 | Chem. Mater. 2018, 30, 5013−5019 | PVA-GD slide ring hydrogels | 82 | 55 | N/A | N/A | ~100% (7) |
| 43 | J. Y. Sun et al., Nature 489, 133–136 (2012). | Hybrid hydrogels (ionic crosslink) | 86 | 8700 | ~160 | ~1500 | 29% (7) |
| 44 | Adv. Mater. 2016, 28, 7178 C7184 | Hybrid DN hydrogels | 79 | 13000 | ~70 | 390 | 36% (4) |
| 42 | ACS Appl. Mater. Interfaces 2019, 11, 5441−5454 | P(AM-co-AA)/Na-alginate/Fe^3+^ hydrogel | 51 | 4800 | ~75 | 400 | 34% (2) |
| 45 | Adv. Mater. 2016, 28, 4884–4890 | Amphiphilic DN hydrogels | 44 | 2850 | N/A | N/A (<600) | 85% (1) |
| 48 | Nature Communications, (2024) 15:1344 | Entangled DN hydrogels (P(AM-co-AMPS) | 90 | 8340 | ~800 | 260 | ~100% (2) |
| 14 | Adv. Mater. 2023, 35, 2301532 | Amphiphilic Entangled DN hydrogels (PEG/PLA) | ~85 | 11000 | ~500 | ~300 | 89% (1) |
| 8 | Kim et al., Science 374, 212–216 (2021) | Entangled SN hydrogels (PAM) | ~80 | 1000 | N/A (<400) | N/A (<450) | ~100% (2.5) |
| 15 | Nature Communications (2024) 15:5896 | Entangled SN hydrogels (PAM) | 70 | 9782 | ~55 | 2000 | 50% (5) |
| 47 | Adv. Funct. Mater. 2021, 31, 2103117 | NC hydrogels (silica nanofiber-PAM) | 82 | ~1500 | ~200 | ~250 | ~100% (1) |
| 46 | Nature Communications (2019) 10:1487 | NC hydrogels (NP-PAA) | NA | ~5500 | N/A (<110) | N/A | ~90% (5) |
| 21 | Adv. Mater. 2022, 34, 2108243 | NC hydrogels (silica nanoparticles-PAM) | 96 | N/A | N/A | N/A | 95% (5) |
| 28 | Nature Materials, 23(1), 131-138 | COF, MS-reinforced hydrogel (crosslinker MBA) | 67 | 3 | ~300 | 750 | ~100% (20) |
| 28 | Nature Materials, 23(1), 131-138 | COF, MS-reinforced hydrogel (crosslinker PEGDA 600) | 67 | 5 | ~150 | 1400 | ~100% (20) |
| **This work** | **This work** | Nanoscale COF nanoconfinement | 67 | 14700 | 458 | 2156 | ~100% (20) |


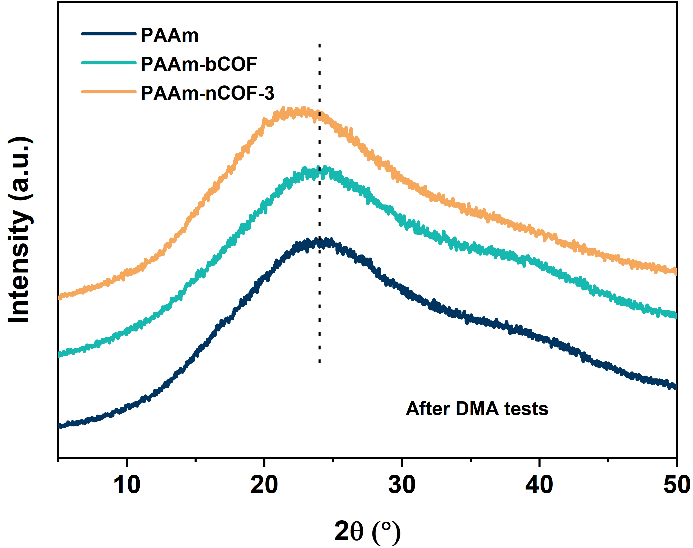


**Figure S18.** PXRD curves of polymers after DMA tests.


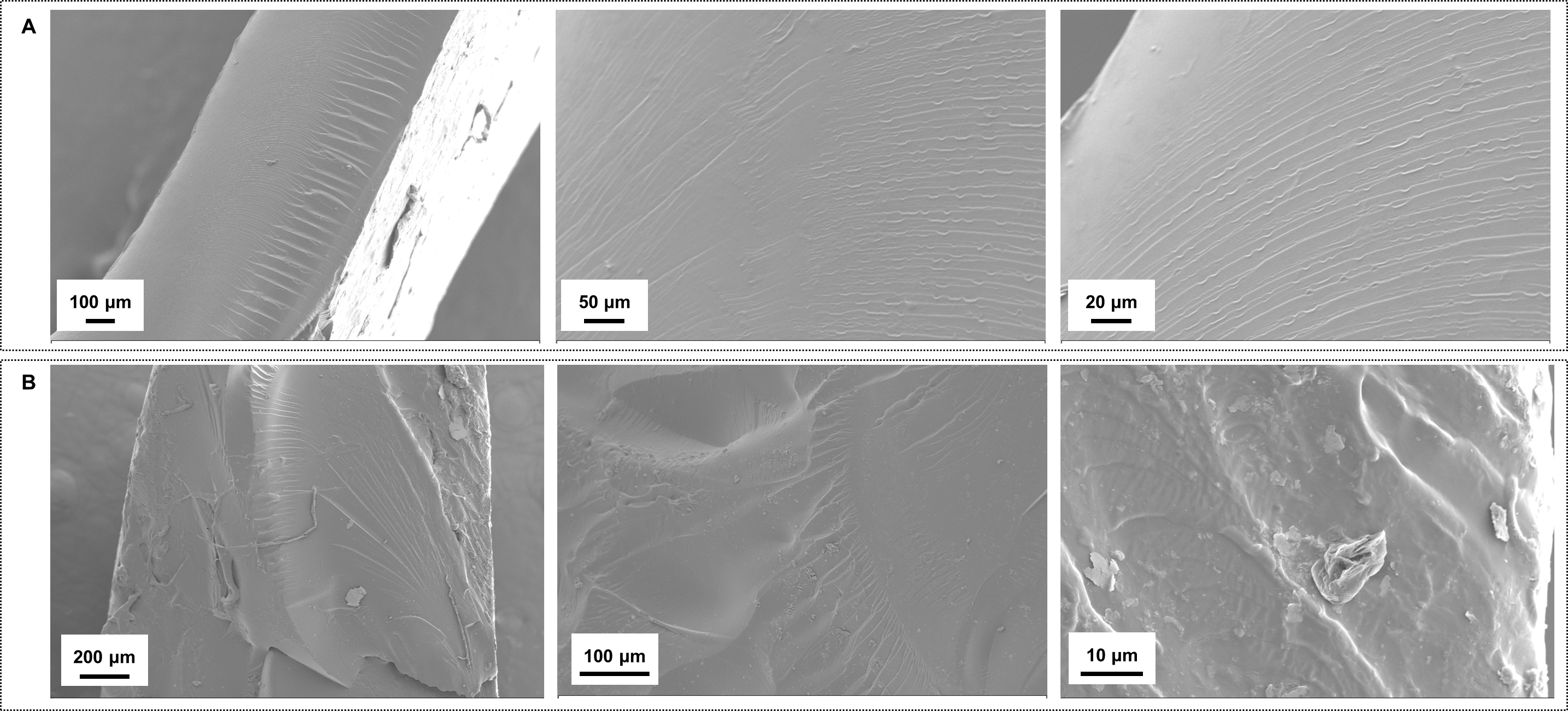


**Figure S19.** Supplemantary SEM images of (A) PAAm-nCOF-3 and (B) PAAm-bCOF with different resolutions.


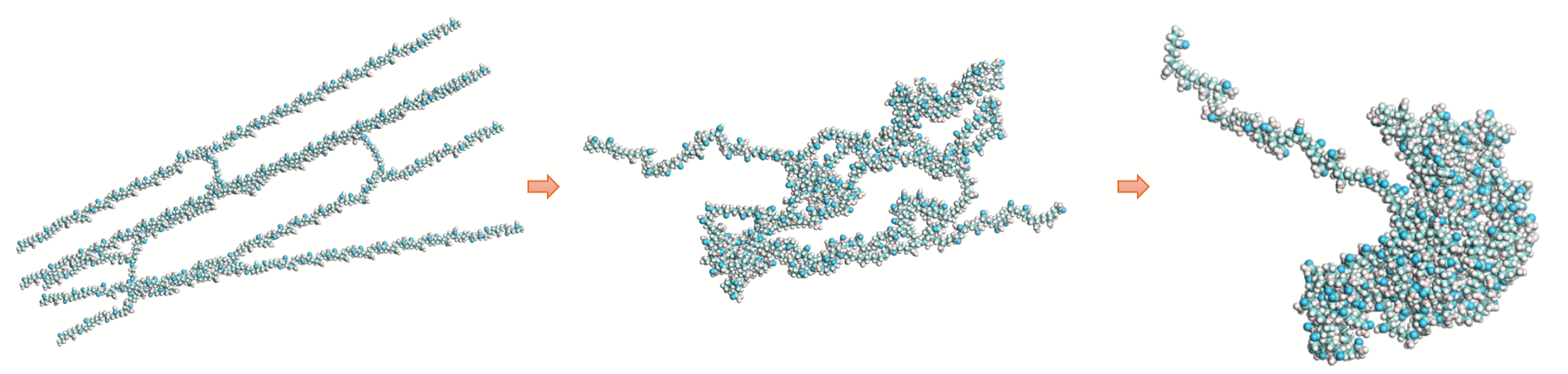


**Figure S20.** Simulation results of polymer chain entanglements.

**Note**: From left to right, the process of entanglement formation driven by the interaction between polymer chains is shown.


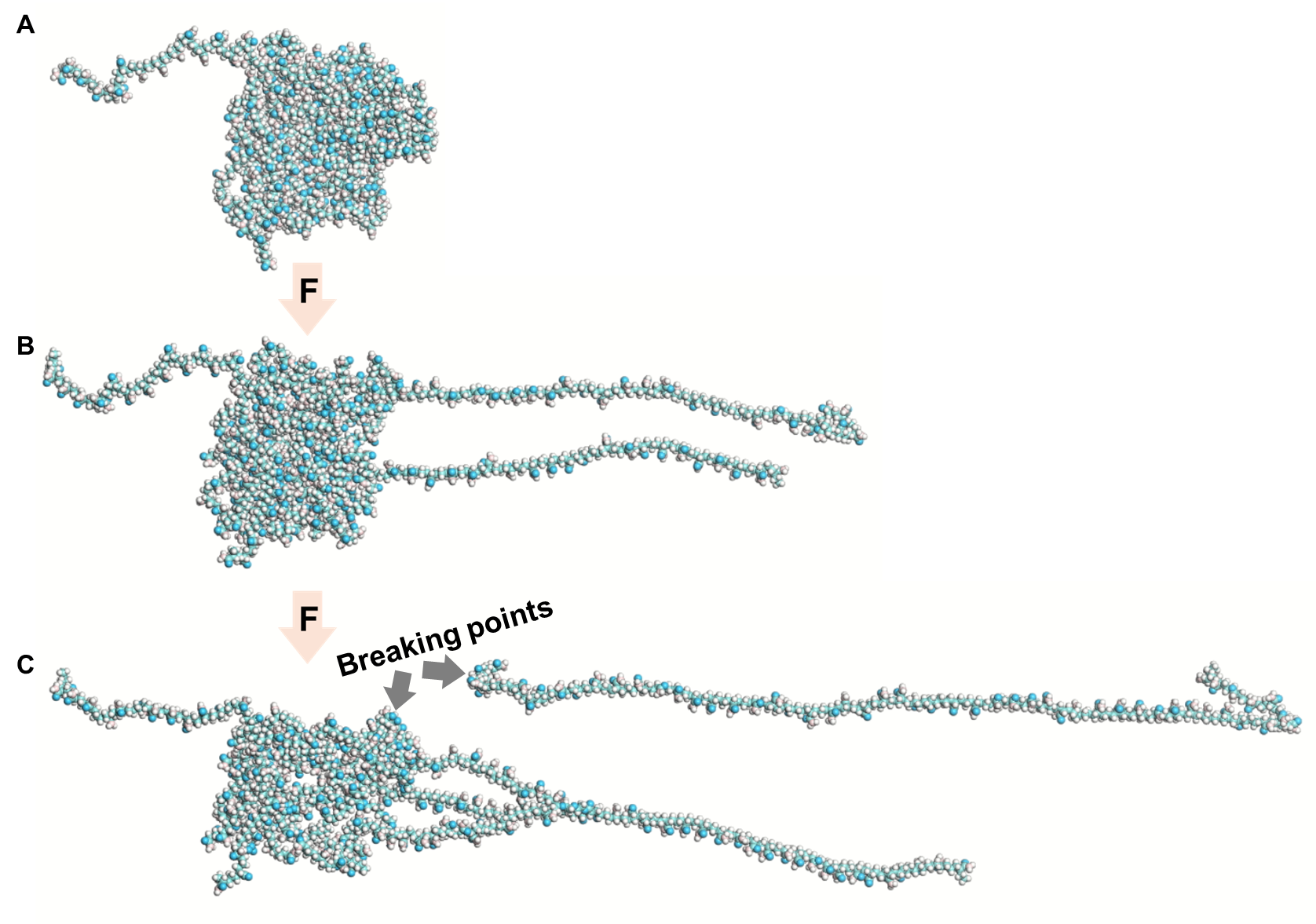


**Figure S21.** Mechanical property simulations of entangled PAAm without nanoscale COF.

**Note**: From panel A to B and C, it is illustrated how the entangled polymer chains evolve during the stretching process until chemical bond breakage occurs, leading to material failure. The highest pull-out energy recorded for this system was 1381.333 kcal/mol.


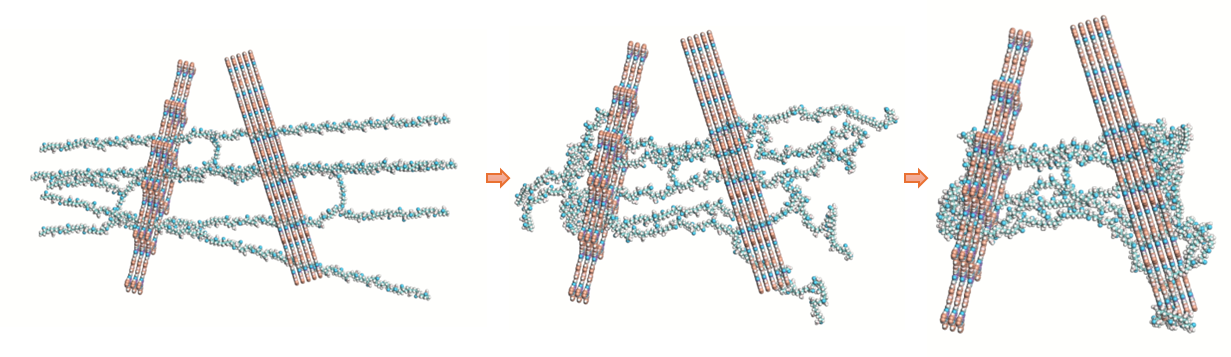


**Figure S22.** Simulation results of polymer chains entanglements formed by threading through pore channels of nanoscale COF.

**Note**: From left to right, the process of entanglement formation is shown through the threading of the pore channels of nanoscale COF and the interface interaction between polymer chains and the COF.


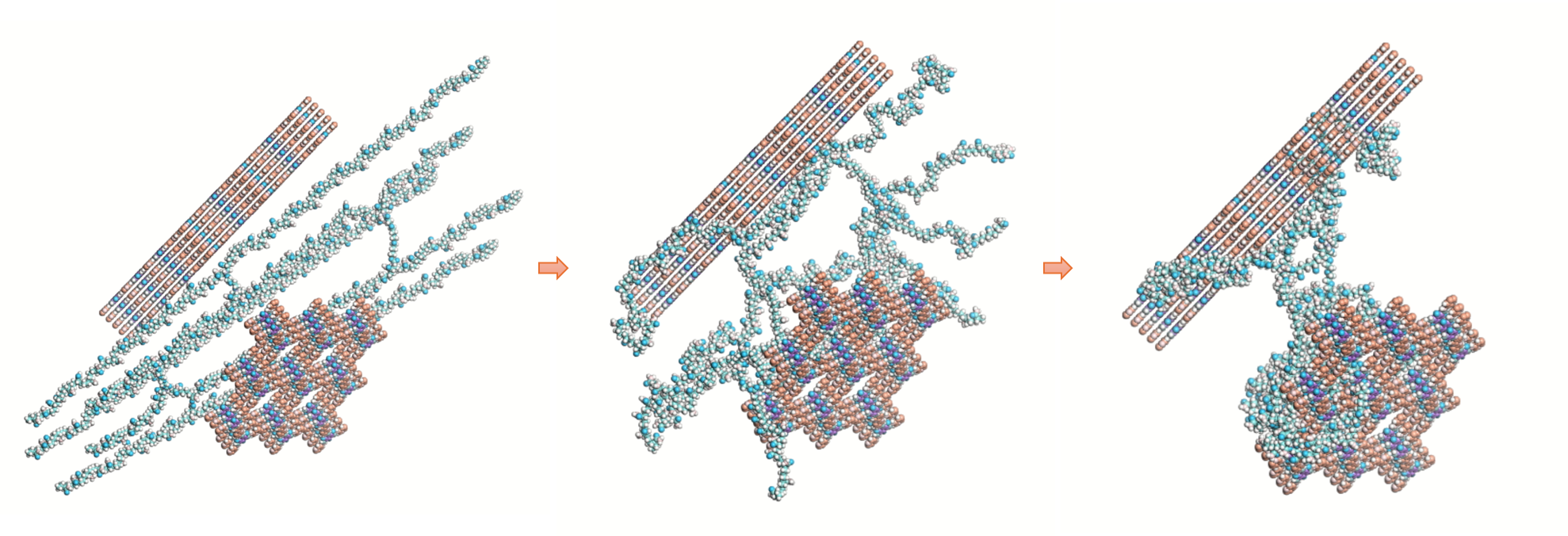


**Figure S23.** Simulation results of polymer chain entanglements formed solely by interface interaction with nanoscale COF (without polymer threading through COF pore channels).

**Note**: From left to right, the process of entanglement formation is illustrated, driven by interface interactions between polymer chains and the COF.

**
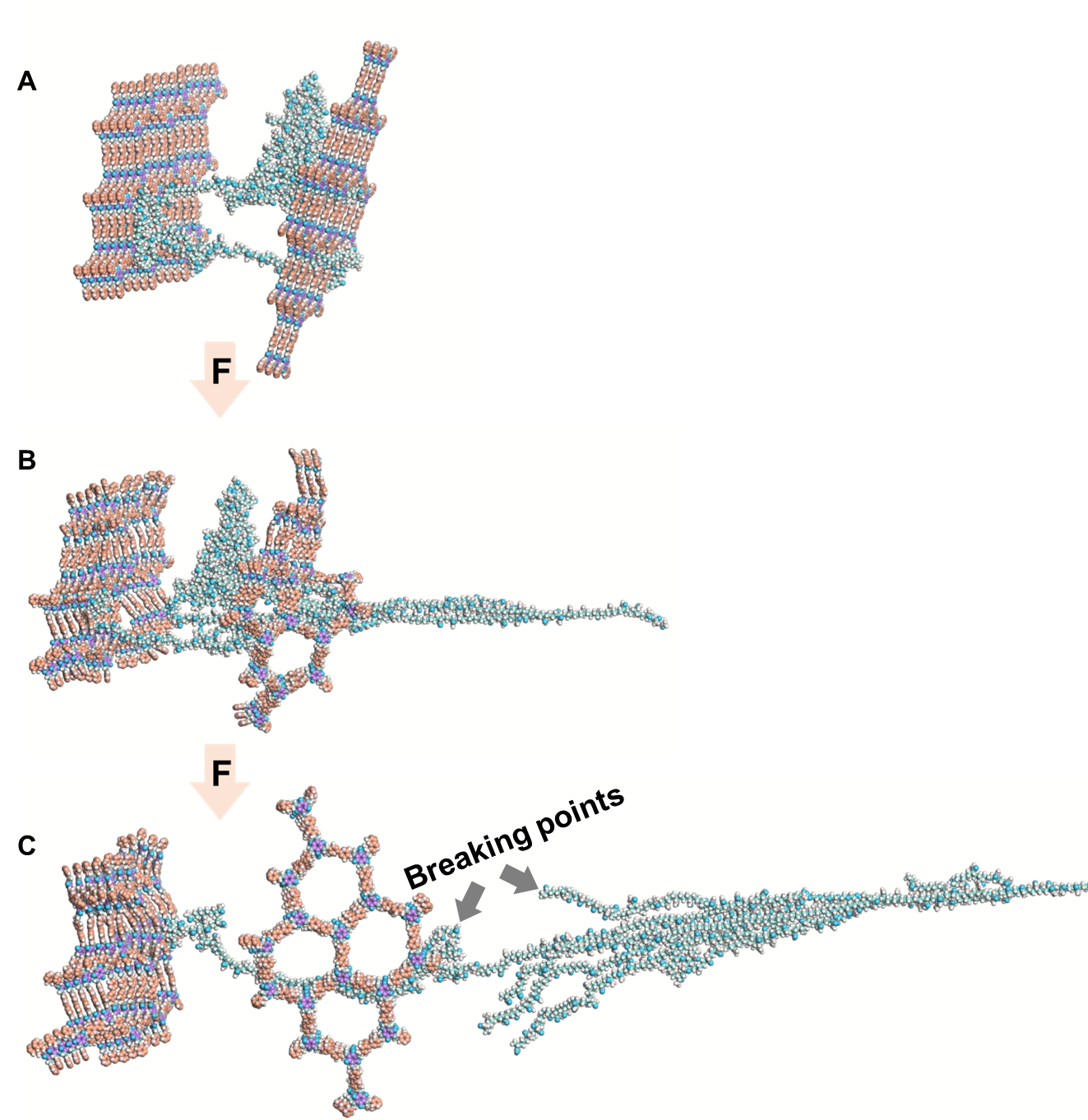
**

**Figure S24.** Mechanical property simulations of PAAm entangled with nanoscale COF, without threading through the pore channels of the COF.

**Note**: From panels A to B and C, it is illustrated how entangled polymer chains, which interact interfacially with nanoscale COFs, evolve during the stretching process until chemical bond breakage occurs under the same applied force, ultimately leading to material failure. The highest pull-out energy recorded was 4,483.894 kJ/m.

**
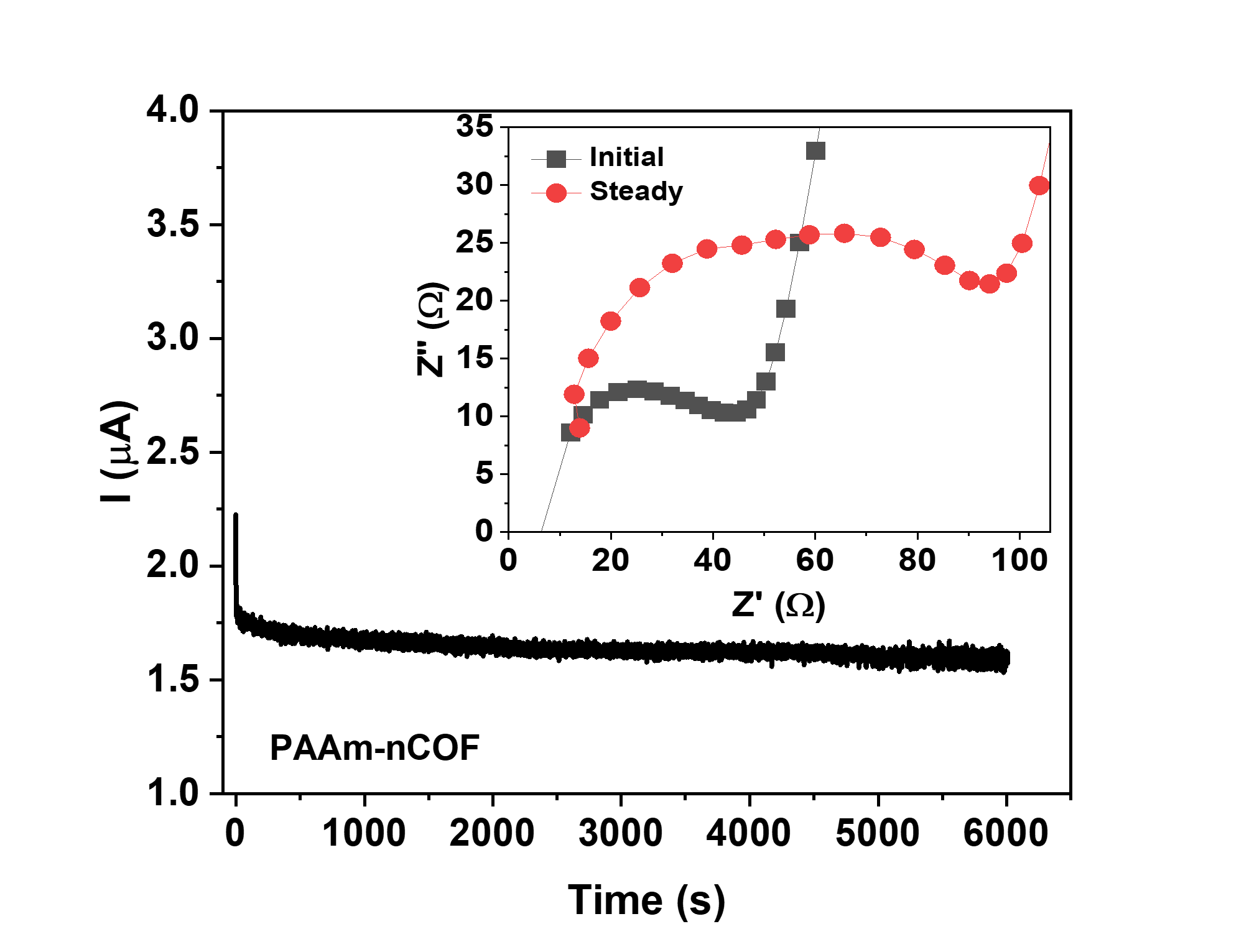
**

**Figure S25.** Chronoamperometry plots of PAAm-nCOF hydrogel electrolyte (inset: EIS plots before and after polarization).

**Figure S26**. Charge-discharge curves at at specific cycle numbers of the Zn| PAAm-nCOF |NVO coin cell at 1A g^-1^.

**Figure S27**. Final charging-discharging curve of full cell assembled with PAAm electrolyte. (Voltage (Y-axis, V) plots to specific capacity (X-axis, mAh/g)).

**Figure S28**. Another set of data: long cycle performance of full cell Zn||NVO assembled hydrogel electrolyte PAAm-nCOF at 1 A/g, compared with the batteries assembled with hydrogel electrolyte PAAm and LE.


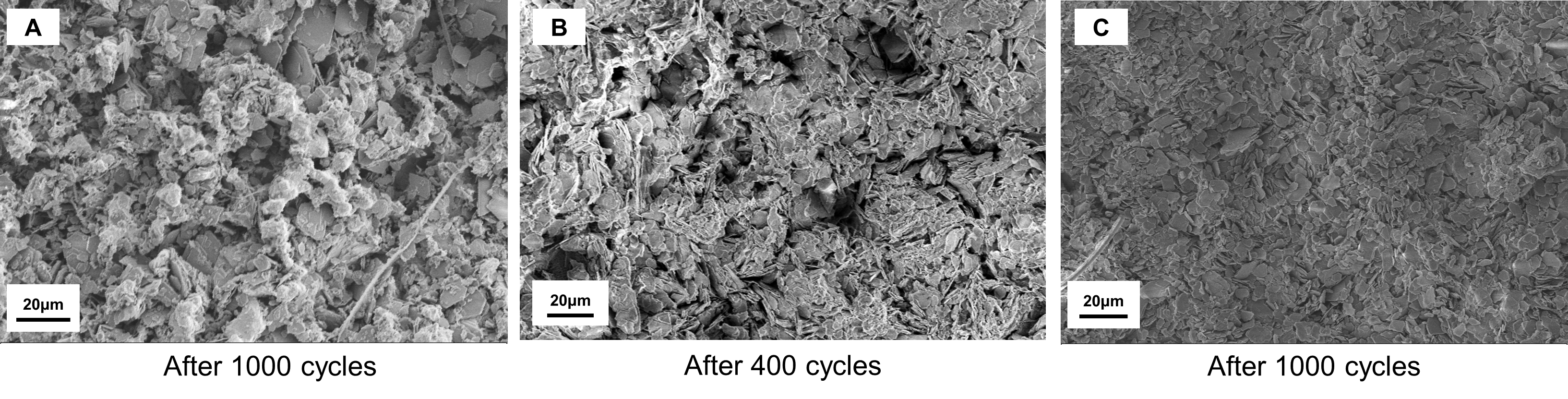


**Figure S29.** SEM images of anode electrodes in full-cell Zn||NVO after long-cycle testing, assembled with (A) LE, (B) PAAm electrolyte, and (C) PAAm-nCOF electrolyte.

| Reference/System | Gel system | Role of COF functional groups | Ionic conductivity | Cathode | Current density | Maximum cycle number | Capacity stability |
| --- | --- | --- | --- | --- | --- | --- | --- |
| This work (PAAm-nCOF) | PAAm-nCOF hydrogel | Mechanical reinforcement; toughness and puncture resistance; uniform Zn deposition | ~16  mS·cm^-1^ | NVO | 1 A g⁻¹ | 1000 cycles | 89% retention |
| Nano Lett. 2024 (10.1021/acs.nanolett.4c03227) | COF-PVA gel electrolyte | Zn^2+^ adsorption and regulation of uniform Zn deposition | ~23.4 mS·cm^-1^ | PANi | 1C | 1000 cycles | ~70-80% retention |
| TCOF-S-Gel, 2023  (Angew. Chem. 2023, 135, e202312020) | Sulfonated COF-functionalized hydrogel | Single-ion Zn^2+^ conduction | ~27.2 mS·cm^-1^ | MnO_2_ | 1C (~1 A g⁻¹) | 1400 cycles | ~96% retention |
| Electrochim. Acta 2024 (10.1016/j.electacta.2024.145059) | Gel electrolyte with COF interlayer | Zn surface stabilization; dendrite suppression | Not applicable | I_2_ | 3C | 650  cycles | ~95% retention |
| Nanoscale 2023 (10.1039/D3NR00898C) | COF-based interfacial layer | Interfacial regulation and Zn protection | Not applicable | MnO_2_ | 0.25 A g^-1^ | 1000 cycles | <80% retention |
| Adv. Energy Mater., 2024 (Adv. Energy Mater. 2024, 14, 2403030) | Artificial Interphase Layer | Polar functional sites regulate Zn^2+^ deposition, homogenize ion flux, suppress side reactions | Not applicable | Activated carbon (Zn-ion capacitors) | 5 A g^-1^ | 100000 cycles | ~95% retention |
| Adv. Funct. Mater., 2026 (10.1002/adfm.202528150) | COF-based hydrogel electrolyte | Janus architecture regulates ion transport pathways and stabilizes electrode/electrolyte interface | ~37 mS cm^-1^ | NVO | 2 A g^-1^ | ~1000 cycles | ~79% |
| Angew. Chem. Int. Ed., 2025 (Angew. Chem. Int. Ed. 2025, 64, e202424184) | COF membrane interphase | Pyridine-N sites regulate interfacial water structure and accelerate Zn^2+^ desolvation | Not applicable | MnO_2_ | 2 A g^-1^ | ~2000 cycles | ~76% |

Table S2. Performance Comparison of COF-Related Hydrogel Electrolytes for Zn Batteries.

## Supporting References

[1] G. Kresse, J. Hafner, "Ab initio molecular-dynamics simulation of the liquid-metal–amorphous-semiconductor transition in germanium", *Physical Review B*, 49 (1994): 14251.

[2] G. Kresse, J. Furthmüller, "Efficiency of ab-initio total energy calculations for metals and semiconductors using a plane-wave basis set", *Computational materials science*, 6 (1996): 15-50.

[3] J.P. Perdew, K. Burke, M. Ernzerhof, "Generalized gradient approximation made simple", *Physical review letters*, 77 (1996): 3865.

[4] P.E. Blöchl, "Projector augmented-wave method", *Physical review B*, 50 (1994): 17953.

[5] S. Grimme, J. Antony, S. Ehrlich, H. Krieg, "A consistent and accurate ab initio parametrization of density functional dispersion correction (DFT-D) for the 94 elements H-Pu", *The Journal of chemical physics*, 132 (2010).

[6] G. Henkelman, B.P. Uberuaga, H. Jónsson, "A climbing image nudged elastic band method for finding saddle points and minimum energy paths", *The Journal of chemical physics*, 113 (2000): 9901-9904.

[7] A.A. Samoletov, C.P. Dettmann, M.A. Chaplain, "Thermostats for “slow” configurational modes", *Journal of Statistical Physics*, 128 (2007): 1321-1336.

[8] H. Sun, "COMPASS: an ab initio force-field optimized for condensed-phase applications overview with details on alkane and benzene compounds", *The Journal of Physical Chemistry B*, 102 (1998): 7338-7364.
